# Lactylation-mediated remodelling of the breast cancer microenvironment: single-cell multidimensional analysis and prognostic model construction

**DOI:** 10.3389/fimmu.2026.1747043

**Published:** 2026-05-13

**Authors:** Jiaxin Chen, Yixue Hao, Yinghua Feng, Yongjing Dai, Haoyuan Shi, Li Zhu

**Affiliations:** 1 Department of Oncology, Chinese PLA General Hospital, Beijing, China; 2 Department of Oncology, The Second Affiliated Hospital of Anhui Medical University, Hefei, China; 3 Department of Epidemiology, School of Public Health, Shanxi Medical University, Taiyuan, Shanxi, China; 4 Senior Department General Surgery, Chinese PLA General Hospital, Beijing, China; 5 Fujian Provincial Key Laboratory of Brain Aging and Neurodegenerative Diseases, School of Basic Medical Sciences, Fujian Medical University, Fuzhou, Fujian, China

**Keywords:** breast cancer, lactylation, tumour microenvironment, prognostic model, single-cell RNA sequencing

## Abstract

**Background:**

The breast cancer tumour microenvironment (TME) exhibits marked cellular and metabolic heterogeneity that contributes to disease progression and therapeutic resistance. Lactylation, a lactate-derived post-translational modification, has emerged as a potential link between metabolic reprogramming and tumour-associated transcriptional and immune changes. However, its cell-type distribution and clinical relevance in breast cancer remain incompletely defined.

**Methods:**

We integrated 26 scRNA-seq samples spanning ER+, HER2+, and triple-negative breast cancer (TNBC), comprising 98,572 cells. A literature-curated lactylation-related transcriptional module score was calculated using AddModuleScore, and epithelial cells were stratified into high- and low-score states for downstream analyses. Candidate genes were prioritised by integrating single-cell differential expression, a curated lactylation-related gene pool, and tumour-associated expression changes in TCGA-BRCA. A 14-gene prognostic model was developed using LASSO-Cox regression in TCGA-BRCA and externally validated in GSE20685 and METABRIC. Additional analyses evaluated associations with immune infiltration, somatic alterations, and predicted drug sensitivity. Functional relevance was explored through CALR knockdown in breast cancer cells and xenograft assays.

**Results:**

Single-cell analysis identified six major cell populations and revealed marked subtype-related heterogeneity. Lactylation-associated transcriptional activity was highest in epithelial cells, particularly in TNBC, and was associated with pathways related to immune response, cell cycle, and metabolic adaptation. High-score epithelial states showed enhanced epithelial–fibroblast–myeloid communication and were linked to an immune-modulatory microenvironment with partial immune-suppressive features. The 14-gene signature stratified patients into significantly different prognostic groups in TCGA-BRCA and retained prognostic value in GSE20685 and METABRIC. Internal resampling suggested modest but reproducible discrimination, although measurable optimism was observed in the training cohort. High-risk tumours were associated with distinct immune/stromal patterns, whereas mutation-frequency differences and tumour mutation burden did not show robust group differences after statistical correction. CALR knockdown suppressed proliferation, induced G1-phase arrest and apoptosis, and reduced xenograft growth.

**Conclusions:**

This study defines a lactylation-associated transcriptional programme in breast cancer and links it to epithelial-state heterogeneity, microenvironmental remodelling, and patient prognosis. The proposed 14-gene signature may provide a transcriptome-based framework for risk stratification, but the findings should be interpreted cautiously because the lactylation score is an indirect surrogate rather than a direct measurement of lactylation itself. Further mechanistic and clinical validation will be required.

## 1 Introduction

Breast cancer is the most common malignancy among women worldwide, and its incidence continues to rise, posing a significant public health challenge (1, 2). Despite substantial advances in surgery, radiotherapy, chemotherapy, endocrine therapy, targeted therapy, and immunotherapy, the prognosis for patients with advanced or metastatic disease remains poor. This outcome largely reflects the marked molecular heterogeneity and complex tumour microenvironment (TME) characteristic of breast cancer (3). However, traditional clinical classifications and pathological indicators still have substantial limitations in predicting patient risk and guiding individualised treatment (4–6). Therefore, exploring the molecular mechanisms of breast cancer, identifying novel prognostic markers, and establishing precise molecular-based risk assessment tools are of great significance for improving its clinical management.

Metabolic reprogramming is a defining feature of cancer biology and represents a key adaptive mechanism that allows tumour cells to thrive in a nutrient- and oxygen-limited microenvironment (7–9). Even under normoxic conditions, breast cancer cells preferentially rely on glycolysis for energy production—a phenomenon known as the Warburg effect (10). This metabolic pattern not only supplies the energy and intermediate metabolites necessary for rapid proliferation but also results in substantial lactate accumulation in the TME (10, 11). Beyond contributing to extracellular acidification, lactate functions as a bioactive signalling molecule that regulates cell proliferation, migration, invasion, and immune evasion (12). Studies have demonstrated that lactate inhibits the activity of CD8^+^ T cells and natural killer cells while promoting the polarisation of regulatory T cells and M2-type macrophages, thereby creating a microenvironment conducive to tumour immune escape (13, 14). Moreover, lactate promotes angiogenesis and stromal remodelling, further underscoring its multifaceted role in cancer progression (15, 16).

Recently, lactate-derived lysine lactylation has been recognised as a novel post-translational modification that extends the biological impact of lactate beyond its role in metabolism. Lactylation modifies not only histone proteins but also non-histone proteins, thereby influencing chromatin status, gene transcription, metabolic enzyme activity, and signal transduction (17). Histone lactylation has been shown to activate gene expression programmes in macrophages, modulating immune responses and inflammation. Emerging evidence also suggests that lactylation contributes to tumour cell proliferation, maintenance of stemness, invasion, therapy resistance, and immune escape—implicating it as a potential driver of breast cancer progression and a promising prognostic biomarker (18, 19).

Despite growing interest in lactate metabolism and lactylation, their global patterns and functional relevance in breast cancer remain poorly characterised (20, 21). Existing studies have primarily focused on individual enzymes or isolated pathways, offering limited insight into lactylation-associated transcriptional networks and their clinical implications (22). Moreover, how lactate-related activity varies across molecular subtypes and individual patients has not been systematically explored. Addressing these gaps requires comprehensive analyses that integrate multi-omics data with single-cell resolution.

Recent studies have increasingly linked metabolic reprogramming to prognosis and immune-context variation in breast cancer, and multi-cohort modelling frameworks have been used to integrate metabolism-related signatures with survival and immune-response prediction (23, 24). At the same time, signature-based bioinformatic studies have highlighted the importance of rigorous validation and cross-method immune association analyses. However, lactate metabolism, lactate-associated transcriptional programmes, and true lactylation modification biology should not be considered interchangeable concepts. In this context, our study was designed to evaluate a literature-curated lactylation-related transcriptional signature as a transcript-level surrogate of lactylation-associated biology rather than as a direct measurement of protein lactylation itself. We therefore combined single-cell transcriptomics, bulk expression data, and experimental validation to characterise lactylation-associated states in breast cancer, construct a prognostic model, and explore their links to the tumour microenvironment in a cautious, association-based framework.

## 2 Materials and methods

### 2.1 Data acquisition and preprocessing

In the training cohort, transcriptomic expression profiles and clinical data were obtained from TCGA-BRCA, including 1,118 breast cancer samples and 113 normal breast tissue samples (25). Differential expression analysis between tumour and normal tissues was performed using DESeq2, with an adjusted P value < 0.05 and |log_2_FC|≥0.5 as the screening criteria. Because the TCGA normal samples were limited in number and not paired with the tumour samples, this bulk differential expression step was used primarily for candidate prioritization rather than as a definitive representation of subtype- or purity-adjusted tumour-specific effects.

Candidate lactylation-related genes were curated from previously published lactylation-focused studies (26–28). These genes were further cross-referenced with gene sets retrieved from the GSEA database, and duplicated gene symbols were removed to generate a literature-curated lactylation-related reference gene pool comprising 414 genes. To assess the robustness of the single-cell findings, we additionally performed a sensitivity analysis using an independent 22-gene lactylation-related panel derived from a published breast cancer study (29). This external panel was analysed using the same AddModuleScore workflow as the primary panel. The full gene list and source information are provided in **Supplementary Table 1**.

For external validation, GSE20685 was used as an independent validation cohort rather than as part of the tumour-normal differential expression step (30). The processed GSE20685 expression matrix was obtained from GEO. Probe identifiers were mapped to gene symbols using the hgu133plus2.db annotation package, probes without valid gene symbols were removed, and when multiple probes mapped to the same gene, the probe with the highest average expression was retained. GSE20685 was processed independently rather than merged with TCGA for direct batch correction, and the TCGA-derived coefficients were directly applied to the overlapping genes without model refitting. In addition, the independent METABRIC cohort with complete survival information was used as a second external validation dataset, and the same TCGA-derived risk-score formula was directly transferred without refitting.

### 2.2 Single-cell RNA-seq analysis

Single-cell RNA sequencing (scRNA-seq) data were obtained from the GSE176078 dataset (31). All downstream analyses were performed in R using the Seurat package (version 5.0.1) (32). During preprocessing, cells with fewer than 200 or more than 10,000 detected genes, as well as those with mitochondrial gene expression exceeding 25%, were excluded to remove low-quality cells. Raw count matrices and sample metadata were used to construct a Seurat object. To enable cross-sample integration, the RNA assay was split into sample-specific layers according to orig.ident. The data were then normalized using the NormalizeData function, and 3,000 highly variable genes were identified using FindVariableFeatures. Scaled expression values were generated using ScaleData, followed by principal component analysis with RunPCA. Cross-sample integration was performed in Seurat v5 using the IntegrateLayers function with the RPCAIntegration method, and the integrated representation was stored as integrated.rpca. Cell-cell neighborhood graph construction and unsupervised clustering were subsequently performed using the FindNeighbors and FindClusters functions based on the first 20 dimensions of the integrated.rpca reduction. UMAP visualization was generated using the same reduction with the RunUMAP function. One low-confidence cluster (cluster 26) was excluded from downstream analyses. Cell-type annotation was performed using canonical marker genes in combination with an automated marker-based annotation procedure, followed by manual review and refinement according to published references (31, 33). Ultimately, six major cell populations were identified: T cells (CD3D, CD3E, CD3G, CD2, CD7, NKG7); myeloid cells (CD68, CD163, CD14, SPP1); B cells (MS4A1, CD79A, CD79B, MZB1, IGKC, JCHAIN); fibroblasts (COL1A1, COL1A2, DCN); epithelial cells (EPCAM, SCGB2A2, CD24, KRT8, KRT7, KRT19); and endothelial cells (CLDN5, FLT1, PECAM1, RAMP2, CDH5, VWF, PTPRB). Post-integration sample distributions were visually examined using UMAP and subtype-stratified visualizations to assess cross-sample mixing.

To estimate lactylation-associated transcriptional activity at the single-cell level, Seurat::AddModuleScore was applied using the literature-curated lactylation-related reference gene set described above. After matching the pooled candidate genes to the scRNA-seq expression matrix and removing unmatched genes, the retained genes were used for module-score calculation. The final gene set used for scoring is provided in **Supplementary Table 1**. Based on the module score within epithelial cells, cells were stratified into high-score and low-score states for downstream analyses. In the primary analysis, epithelial cells were dichotomized according to the median module score. To assess the robustness of this grouping strategy, the downstream analyses were repeated using tertile- and quartile-based definitions of high-score and low-score cells. To provide orthogonal support for the immune-related interpretation of the high-score state, we additionally evaluated exhaustion- and Treg-associated signatures in T cells and an M2-like signature in myeloid cells.

### 2.3 GO and KEGG enrichment analysis and intercellular communication analysis

The biological processes underlying the enrichment of differentially expressed genes (DEGs) were explored using gene ontology (GO) and KEGG pathway analyses. The degree of each cell type was analysed using the clusterProfiler::gseGO (Version 4.8.1) and clusterProfiler::gseKEGG packages. Functional enrichment analysis and cell marker annotation enrichment (34) were performed using the org.hs.eg.db annotation package (Version 3.17.0). Intercellular communication analysis was performed using the CellChat R software package (Version 2.1.2) (35). Specifically, only receptors and ligands expressed in more than 0.1% of the cells are analysed; if neither can be detected, it is considered that there is no communication.

### 2.4 Establishment of a prognostic risk scoring model based on lactylation-related genes

Least absolute shrinkage and selection operator (LASSO) Cox regression analysis was performed using the R packages “survival” and “glmnet” to identify Lactylation-related genes significantly associated with overall survival (OS) in breast cancer patients. Based on the regression coefficients, a risk score for each patient was calculated using the formula: Risk score = Σ (coefficient_i × expression_i). A protein–protein interaction (PPI) network was constructed using the STRING database (https://string-db.org/). Patients were then stratified into high- and low-risk groups according to the median risk score. Based on these genes and their regression coefficients, the risk score for each patient was calculated as follows: Risk Score = -0.137 × HIBCH + 0.074 × FBP1 + 0.013 × ARID3A − 0.126 × CALR − 0.160 × MAGOHB + 0.003 × GAPDH − 0.089 × PGK1 + 0.367 × MKI67 − 0.031 × PTMA − 0.051 × RPL5 + 0.067 × RPS23 − 0.031 × TYMP − 0.140 × GK − 0.013 × COX6B1. Kaplan–Meier survival analysis was performed using the R package “survival” to compare overall survival between the two groups. Time-dependent ROC curves were generated using the R package “timeROC” to assess the predictive performance of the model, and visualisations were created with “ggplot2”. Furthermore, a nomogram integrating risk scores and clinical features was constructed using the R packages “rms” and “survival”. Calibration curves were plotted to compare predicted and observed survival probabilities, thereby validating the model’s accuracy and clinical applicability. A prognostic nomogram was constructed by integrating the risk score with selected clinicopathological variables. Only patients with complete information for the included variables were retained for nomogram analysis. Model performance was evaluated using calibration analysis, concordance index (C-index), and decision curve analysis (DCA) to assess potential clinical utility. To further examine the risk of overfitting, additional internal validation analyses were performed in the TCGA cohort, including repeated 10-fold cross-validation to assess model stability and bootstrap resampling to estimate optimism in the training cohort. The TCGA-derived risk-score formula was then directly applied, without refitting, to the external GSE20685 and METABRIC cohorts for independent validation.

### 2.5 Somatic mutation and immune infiltration analysis

Somatic mutations were merged across TCGA-BRCA MAF files and analysed using maftools (version 2.14.0). Mutation frequency differences between risk groups were assessed using Fisher’s exact test with FDR correction implemented in the maftools package. Tumor mutation burden (TMB) was calculated as the number of non-synonymous mutations per megabase and compared between groups using the Wilcoxon rank-sum test. Non-silent mutations in the high- and low-risk groups were visualised as oncoplots. Immune cell fractions were deconvolved by CIBERSORT (LM22 signature matrix) from log2(TPM + 1)-transformed expression data using a custom R script, retaining samples with P < 0.05. The estimated proportions were compared between risk groups using the Wilcoxon test, and visualised with ggplot2. Immune deconvolution was performed using CIBERSORT (LM22, 1,000 permutations, quantile normalization disabled for RNA-seq input), and only samples with deconvolution P < 0.05 were retained. MCP-counter was additionally applied as an alternative method to assess robustness of the immune/stromal findings.

### 2.6 Predictive analysis of drug sensitivity

OncoPredict (version 2.2.1) is used to predict the IC50 value of GDSC2 drugs in TCGA-BRCA samples. The training data (GDSC2 expression and response) were batch-corrected using ‘sva::ComBat’ (version 3.48.0) with TCGA data. Remove low-variance genes (>80% removal) and apply the power conversion phenotype. Predictive sensitivity was compared between risk groups using the Wilcoxon test (P<0.05), and significant drugs are shown as box plots.

### 2.7 Cell culture, plasmids, RNA oligonucleotides and reagents

Human breast cancer cell lines ZR-751 and MDA-MB-231 were cultured in RPMI-1640 (Gibco, Cat#: 11875093) supplemented with 10% fetal bovine serum (EVERY GREEN, Cat#: 11011-8611) and 1% penicillin-streptomycin (Gibco, Cat#: 15140122). All cell lines were obtained from the American Type Culture Collection (ATCC) and had been previously tested for mycoplasma contamination. Cells were incubated at 37 °C in a humidified atmosphere containing 5% CO_2_.

For CALR knockdown, small interfering RNAs (siRNAs) specifically targeting CALR were synthesised by JTS Scientific. The target sequences were as follows: siCALR#1:5’-GGAUCUACCUCUGGAAGAA-3’,siCALR#2:5’-CCAGAUUGCUACUGGAUAA-3’. The two siRNAs (siCALR#1 and #2) were designed and synthesised (targeting non-overlapping regions), and transiently transfected into cells using Lipofectamine RNAi MAX (Invitrogen, Cat#: 13778150) following the manufacturer’s protocol. Small interfering RNAs (siRNAs) specifically targeting CALR were synthesised by JTS Scientific and transiently transfected into cells using Lipofectamine RNAi MAX (Invitrogen, Cat#: 13778150) following the manufacturer’s instructions. Knockdown efficiency was confirmed by Western blot. Anti-CALR antibody (Rabbit Polyclonal, Cat#: T55353S) was purchased from ABMART. For rescue experiments, cells were transfected with a CALR overexpression plasmid (MiaolingBiology, P97059) using Lipofectamine 3000 (Invitrogen,L3000075) according to the manufacturer’s protocol. For rescue assays, CALR overexpression was introduced following siRNA-mediated knockdown.

### 2.8 Colony formation assay

Cells were seeded at 1000 cells/well in 6-well plates and cultured in DMEM medium supplemented with 10% FBS at 37 °C for 14 days. Colonies were fixed with 4% paraformaldehyde, stained with crystal violet (Beyotime, Cat#: C0121), and counted. All experiments were conducted in triplicate.

### 2.9 Cell proliferation assay

Cell proliferation was quantified using the CCK-8 kit (Dojindo, CK04) following the manufacturer’s instructions. Briefly, cells were seeded into tissue-culture–treated 96-well flat-bottom plates (outer wells filled with sterile PBS to minimise edge effects) at a density empirically optimised for each cell line to remain in logarithmic growth over 7 days (typically 2000 cells per well for adherent lines), with ≥ 3 technical replicates per condition and ≥ three independent biological repeats. Cells were cultured at 37 °C with 5% CO_2_. At days 1, 3, 5, and 7 post-seeding, 10 µL of CCK-8 solution was added to each well containing 100 µL of culture medium (1:10, v/v), and the plate was incubated for two hours in the dark. Absorbance at 450 nm (A450) was recorded using a microplate reader (Thermo Scientific, Multiskan FC).

### 2.10 Western blot

Total protein was extracted using RIPA buffer (Beyotime, Cat#: P0013B) supplemented with protease and phosphatase inhibitors (Beyotime, Cat#: P1045). Protein concentrations were determined using the BCA Protein Assay Kit (Thermo Scientific, Cat#: 23227). Equal amounts of protein (30 μg per lane) were separated by 10% SDS-PAGE and transferred onto nitrocellulose (NC) membranes (Pall, Cat#: 66485). Membranes were blocked in 5% non-fat dry milk (Bio-Rad) in TBST for 1 hour at room temperature, followed by incubation with primary antibodies overnight at 4 °C. The following primary antibodies were used: Anti-CALR (Abmart, Rabbit Polyclonal, Cat#: T55353S,1:1000 dilution), Anti-β-Actin (Abmart, Cat#: T40104S, 1:5000 dilution, used as loading control), Flag-HRP (Sigma,A8592,1:2000). After washing, membranes were incubated for 1 hour at room temperature with HRP-conjugated secondary antibody (Abmart, Cat#: M21008). Signal detection was performed using an ECL substrate (Thermo Fisher Scientific, Cat#: 32106), and chemiluminescent signals were imaged using the ChemiDoc™ XRS+ system (Bio-Rad).

### 2.11 Cell cycle and cell apoptosis analysis

Cell cycle distribution was analysed using the Cell Cycle Detection Kit (Biosharp, Cat. No. BL114A) following the manufacturer’s protocol. Briefly, cells were harvested, washed twice with cold PBS, and fixed in pre-chilled 70% ethanol at 4 °C for two hours to ensure complete permeabilisation. After fixation, cells were washed twice with PBS and incubated with staining buffer containing RNase (1 mg/mL) and propidium iodide (PI, 50μg/mL) at 37 °C for 30 min in the dark. The stained cells were centrifuged, resuspended in PBS, and immediately analysed by flow cytometry (BD FACSCanto II). A minimum of 10,000 events was collected per sample, and the proportions of cells in G_0_/G_1_, S, and G_2_/M phases were quantified using FlowJo software (v10.8.1). Apoptosis was evaluated using the Dead Cell Apoptosis Kit with Annexin V-FITC & Propidium Iodide (Thermo Fisher Scientific, Cat. No. V13242) in accordance with the manufacturer’s instructions. In brief, harvested cells were washed twice with cold PBS and resuspended in 1× Annexin-binding buffer at a density of ~1 × 10^6^ cells/mL. For each 100 μL cell suspension, five μL FITC-Annexin V and one μL PI (100 μg/mL working solution) were added, followed by gentle mixing and incubation for 30 min at room temperature in the dark. Subsequently, 400 μL of 1× Annexin-binding buffer was added, and the samples were kept on ice until analysis. Flow cytometric acquisition was performed within one hour.

### 2.12 Cell line-derived xenograft model

All animal experiments were approved and guided by the Ethics Committee of the Beijing Institute of Biotechnology (approval number IACUC-DWZX-2022-009) and were performed in accordance with the principles of the 3Rs (Reduction, Refinement, and Replacement) and the NIH Guide for the Care and Use of Laboratory Animals. Mice were maintained under specific-pathogen-free (SPF) conditions in a controlled environment (12 h light/12 h dark cycle, 22 ± 2 °C, 40–60% relative humidity) with ad libitum access to food and water. To establish the ZR751 cell-derived xenograft model, six-week-old female BALB/c-nu/nu mice (16–18 g, SPF grade, certification No. SCXK [Beijing] 2019–0010) were obtained from SPF (Beijing) Biotechnology Co., Ltd. (Beijing, China). A total of 1 × 10^6^ 751^shNC^ or 751^shCALR^ cells were resuspended in 100 µL of serum-free DMEM and Matrigel (ABW BIO, China) mixture (1:2, v/v), and injected subcutaneously into the right dorsal flank of each mouse. Tumour growth was monitored every five days for a total of 55 days. Tumour volume (V) was calculated using the standard formula=length×(width)2/2. At the end of the observation period, mice were euthanised, and tumours were excised, weighed, and processed for subsequent histopathological and immunohistochemical analyses. Survival curves were generated using GraphPad Prism 10.

### 2.13 Histology and immunohistochemistry

Xenograft tumours were collected at the endpoint and immediately fixed in 10% neutral-buffered formalin (Thermo Fisher Scientific) overnight at room temperature. Following fixation, the tissues were rinsed once with PBS, transferred to 70% ethanol, and processed for paraffin embedding. Paraffin blocks were sectioned at a four μm thickness and subjected to routine haematoxylin and eosin (H&E) staining for histopathological examination.

For immunohistochemical (IHC) staining, formalin-fixed and paraffin-embedded sections were deparaffinised in xylene, rehydrated through graded ethanol, and treated with 3% hydrogen peroxide (H_2_O_2_) for 15 min to quench endogenous peroxidase activity. Antigen retrieval was performed in 10 mM citrate buffer (pH 6.0) by microwave heating for 30 min. After cooling to room temperature, tissue sections were blocked with 5% bovine serum albumin (BSA) and incubated overnight at 4 °C with the following primary antibodies: anti-CALR (1:500, ab92516, Abcam), anti-Ki-67 (1:200, ab16667, Abcam). The next day, slides were washed and incubated with HRP-conjugated secondary antibodies at room temperature for one hour. The immunoreactive signals were visualised using 3,3′-diaminobenzidine (DAB) substrate, followed by counterstaining with haematoxylin. Images were captured at 400×magnification using an Olympus BX43 microscope. Semi-quantitative analysis of IHC staining was performed using the immunoreactive score (IRS) system. The proportion of positively stained cells was scored as follows: 0 (no staining), 1 (1–10%), 2 (11–50%), 3 (51–80%), and 4 (81–100%). Staining intensity was graded as 0 (no reaction), 1 (weak), 2 (moderate), and 3 (strong). The final IRS was calculated as: IRS = (intensity score) × (percentage score). Each slide was independently evaluated by two experienced pathologists blinded to group allocation, and data were expressed as mean ± SD.

### 2.14 Statistical analysis

All statistical analyses were performed in R (version 4.5.1). Unless otherwise specified, all tests were two-sided, and P<0.05 was considered statistically significant. Continuous variables were compared using Student’s t-test or the Wilcoxon rank-sum test as appropriate, and categorical variables were compared using the chi-square test or Fisher’s exact test where applicable. Survival differences were evaluated using Kaplan-Meier analysis with the log-rank test, and independent prognostic factors were assessed using Cox proportional hazards regression. Data from *in vitro* and *in vivo* experiments are presented as mean ± SD. The specific number of biological replicates for each experiment is indicated in the corresponding figure legends.

## 3 Results

### 3.1 Single-cell transcriptome profiling reveals the heterogeneity and subtype specificity of the breast cancer microenvironment

**Figure 1:**
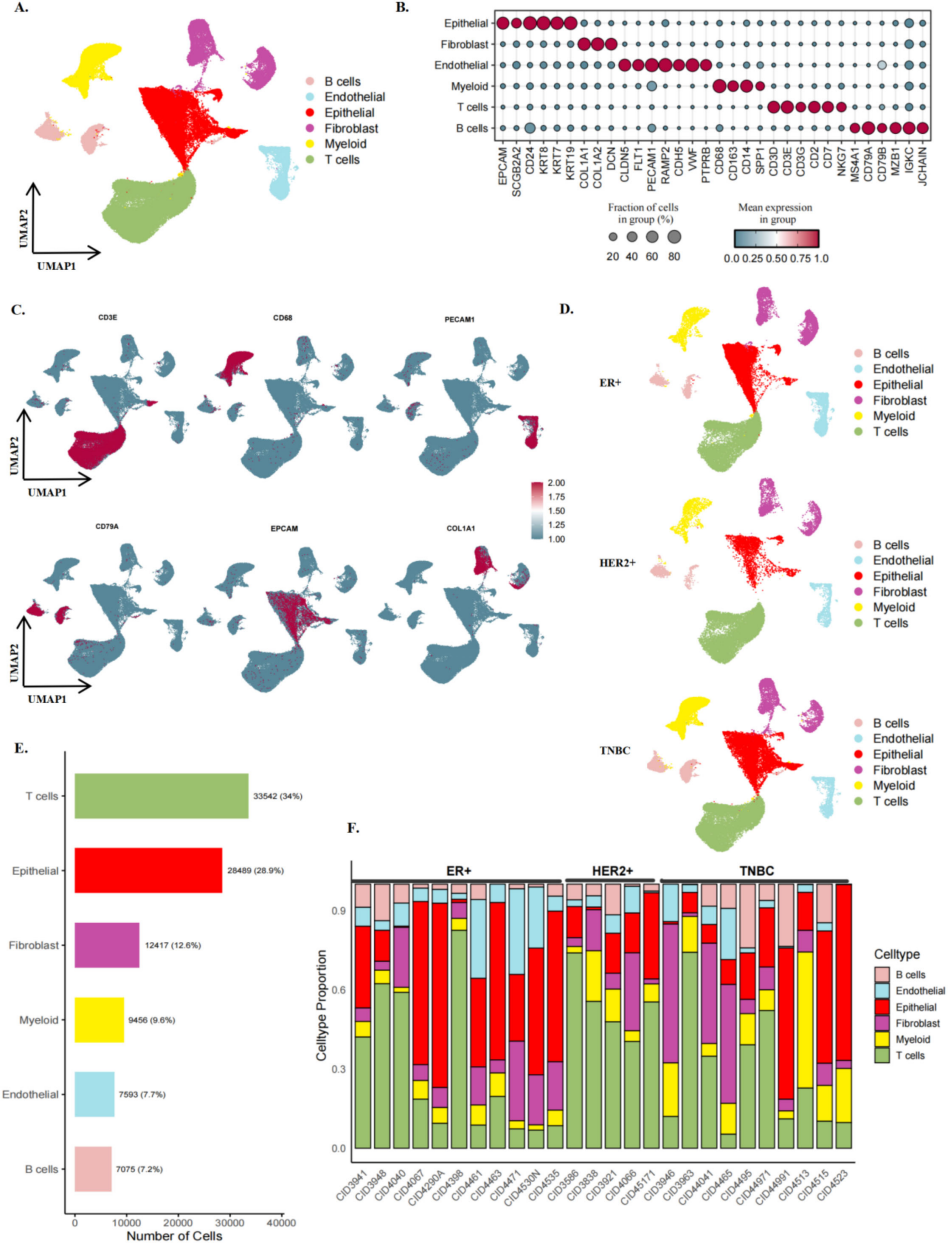
Single-cell transcriptome analysis of breast cancer reveals microenvironmental heterogeneity and subtype variation. **(A)** The UMAP dimensionality reduction map shows the clustering of each cell type. **(B)** Dot plots show the expression levels of marker genes across different cell types, with dot size and colour representing the frequency and intensity of gene expression, respectively. **(C)** The UMAP visualisation displayed the expression and distribution of marker genes, including CD3E, CD68, COL1A1, CD79A, PECAM1, and EPCAM, across different cell types. **(D)** Stratified UMAP views by molecular subtypes (ER+, HER2+, and TNBC), highlighting subtype-specific cell distribution variations. **(E)** Quantitative statistical bar charts of each cell type. **(F)** The stacked bar chart shows the proportions of individual cell types across different patients.

To characterise the single-cell transcriptomic landscape of lactate-related activity in breast cancer, we obtained and analysed 26 samples from public scRNA-seq datasets, covering primary tumour subtypes including ER+, HER2+, and TNBC. After rigorous quality control, 98,572 high-quality cells were retained for downstream analyses. UMAP-based dimensionality reduction revealed distinct cellular clusters in two dimensions, yielding 47 clusters (**Supplementary Figure 1A**). Using Seurat V5 (32), we classified these cells into six major types based on canonical marker genes: epithelial cells, fibroblasts, myeloid cells, B cells, T cells, and endothelial cells (**Figure 1A**). To further characterise the molecular features of each cell population, bubble plots and feature maps were generated to visualise the expression patterns of representative marker genes. T cells were characterized by CD3D, CD3E, CD3G, CD2, and CD7 expression; myeloid cells by CD68, CD14, CD63, and SPP1; B cells by MS4A1, CD79A, CD79B, MZB1, IGKC, and JCHAIN; fibroblasts by COL1A1, COL1A2, and DCN; epithelial cells by EPCAM, SCGB2A2, CD24, KRT8, KRT7, and KRT19; and endothelial cells by CLDN5, FLT1, PECAM1, RAMP2, CDH5, VWF, and PTPRB (**Figures 1B, C**). Hierarchical visualisation based on breast cancer molecular subtypes (ER+, HER2+, TNBC) revealed distinct subtype-specific expression differences (**Figure 1D**). Further analysis of cellular composition showed that T cells and epithelial cells were the predominant populations across all samples, accounting for 33,542 (34.0%) and 28,489 (28.9%) cells, respectively. Fibroblasts, myeloid cells, endothelial cells, and B cells accounted for 12,417 (12.6%), 9,456 (9.6%), 7,593 (7.7%), and 7,075 (7.2%) cells, respectively (**Figure 1E**). Subtype-based analysis revealed significant differences in the relative abundance of epithelial and immune cells (**Supplementary Figure 1B**). At the individual level, HER2+ samples exhibited the lowest proportion of epithelial cells, whereas TNBC samples showed a relatively reduced proportion of endothelial cells. Despite inter-individual and tissue heterogeneity, most samples maintained relatively stable cellular composition patterns, reflecting the unique cellular ecosystem of breast cancer (**Figure 1F**).

### 3.2 A lactylation-associated transcriptional program is linked to immune-stromal communication and microenvironmental remodelling in breast cancer epithelial cells

**Figure 2:**
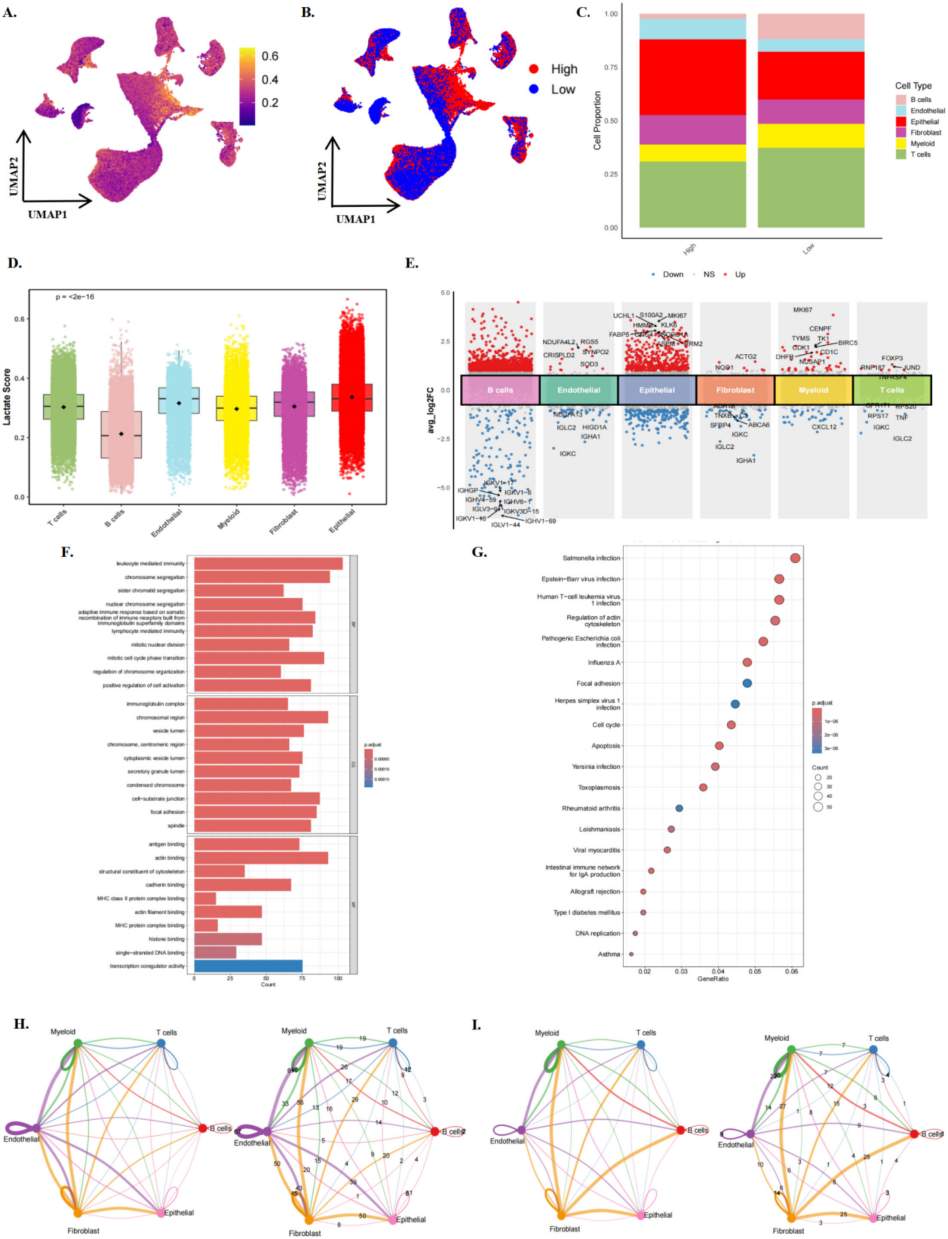
Distribution and correlation analysis of lactylation score in the single-cell microenvironment of breast cancer. **(A)** The UMAP dimensionality-reduction map shows the spatial distribution of Lactylation scores across the overall cell landscape, with colour gradients from low (purple) to high (yellow). **(B)** UMAP view of the high and low gradient distribution of lactylation scores, emphasising the enrichment of specific cell clusters. **(C)** The stacked bar chart compares the proportions of each cell type in the high-lactylation group (High) and the low rating group (Low). **(D)** The box plot shows the statistical distribution of lactylation-related gene expression in different cell types (B cells, T cells, myeloid cells, endothelial cells, fibroblasts, epithelial cells). **(E)** Multiple sets of volcano diagrams show the analysis of differences among various cell types. **(F)** The bar chart shows enrichment of GO pathways associated with lactylation scores. **(G)** The dot plot shows the KEGG enrichment analysis of the association between lactylation score and disease. The dot size indicates the count, and the colour indicates the adjusted p-value, highlighting significant associations (such as p < 0.05). **(H)** Network diagram and weight diagram of intercellular interactions in the high-activity group. **(I)** Network diagram and weight diagram of intercellular interactions in the low-activity group.

We applied the AddModuleScore algorithm to quantify the Lactylation-related gene activity in each cell, revealing that although Lactylation scores were detectable across all cell types, epithelial cells consistently exhibited the highest activity (**Figure 2A**). Based on the median lactylation score, cells were stratified into high- and low-lactylation groups, revealing that high-lactylation activity was predominantly enriched in epithelial cells and fibroblasts (**Figure 2B**). Using the median module score, epithelial cells were divided into high- and low-lactylation-activity groups for downstream analyses. Sensitivity analyses based on alternative tertile- and quartile-based cutoffs yielded broadly consistent patterns in differential expression, pathway enrichment, and intercellular communication, indicating that the major conclusions were not driven by a single threshold definition (**Supplementary Figure 2**). UMAP visualisation of Lactylation activity across molecular subtypes yielded consistent results (**Supplementary Figure 1C**). Further quantitative analysis indicated that epithelial cells accounted for the most significant proportion in the high-activity group, followed by T cells, suggesting that Lactylation-related processes predominantly occur within breast cancer epithelial cells (**Figure 2C**). At the cell-type level, Lactylation scores in epithelial cells were significantly higher than in all other cell types, with B cells exhibiting the lowest activity (p < 2e-16) (**Figure 2D**). Subtype analysis demonstrated that epithelial cells were the main Lactylation-active population, with the highest activity observed in TNBC and the lowest in ER+ samples (**Supplementary Figure 1D**). Differential expression analysis between high- and low-Lactylation activity groups, followed by GO and KEGG enrichment analyses, revealed that genes upregulated in the hyperLactylation group were predominantly enriched in immune response, cell cycle regulation, and transcription factor activation pathways (**Figures 2E–G**). Moreover, intercellular communication network analysis indicated that in the high-Lactylation group, the interaction strength between myeloid cells and T cells, as well as between epithelial cells and fibroblasts, was markedly enhanced. In contrast, in the low-Lactylation group, interactions were relatively sparse and primarily characterised by signalling among fibroblasts, myeloid cells, and B cells. These findings highlight the pivotal role of Lactylation in orchestrating immune–stromal interactions and driving microenvironmental remodelling in breast cancer. To assess robustness, we repeated the scoring analysis using an independent external 22-gene lactylation-related panel reported in breast cancer (29). Although overall cell-type separation was attenuated compared with the broader primary panel, the principal trends were preserved, with epithelial cells remaining among the highest-scoring populations and TNBC epithelial cells showing the strongest enrichment(**Supplementary Figure 2**). We also repeated the downstream analyses using tertile- and quartile-based grouping strategies. Although the magnitude of differential expression varied across cell types, the major functional themes remained broadly similar, particularly with respect to RNA-processing, cell-cycle, and proliferation-associated pathways(**Supplementary Figure 3**). Finally, orthogonal immune-signature analysis showed that the high-score context was associated with increased T-cell exhaustion, whereas the Treg signature was not significantly different and the myeloid M2-like pattern was less concordant(**Supplementary Figures 4A, B**). Taken together, these findings support a more cautious interpretation of the high-score state as an immune-modulatory microenvironment with partial immune-suppressive features, rather than definitive evidence of a fully established immunosuppressive niche.

### 3.3 Construction of a prognostic model related to lactylation based on machine learning

**Figure 3:**
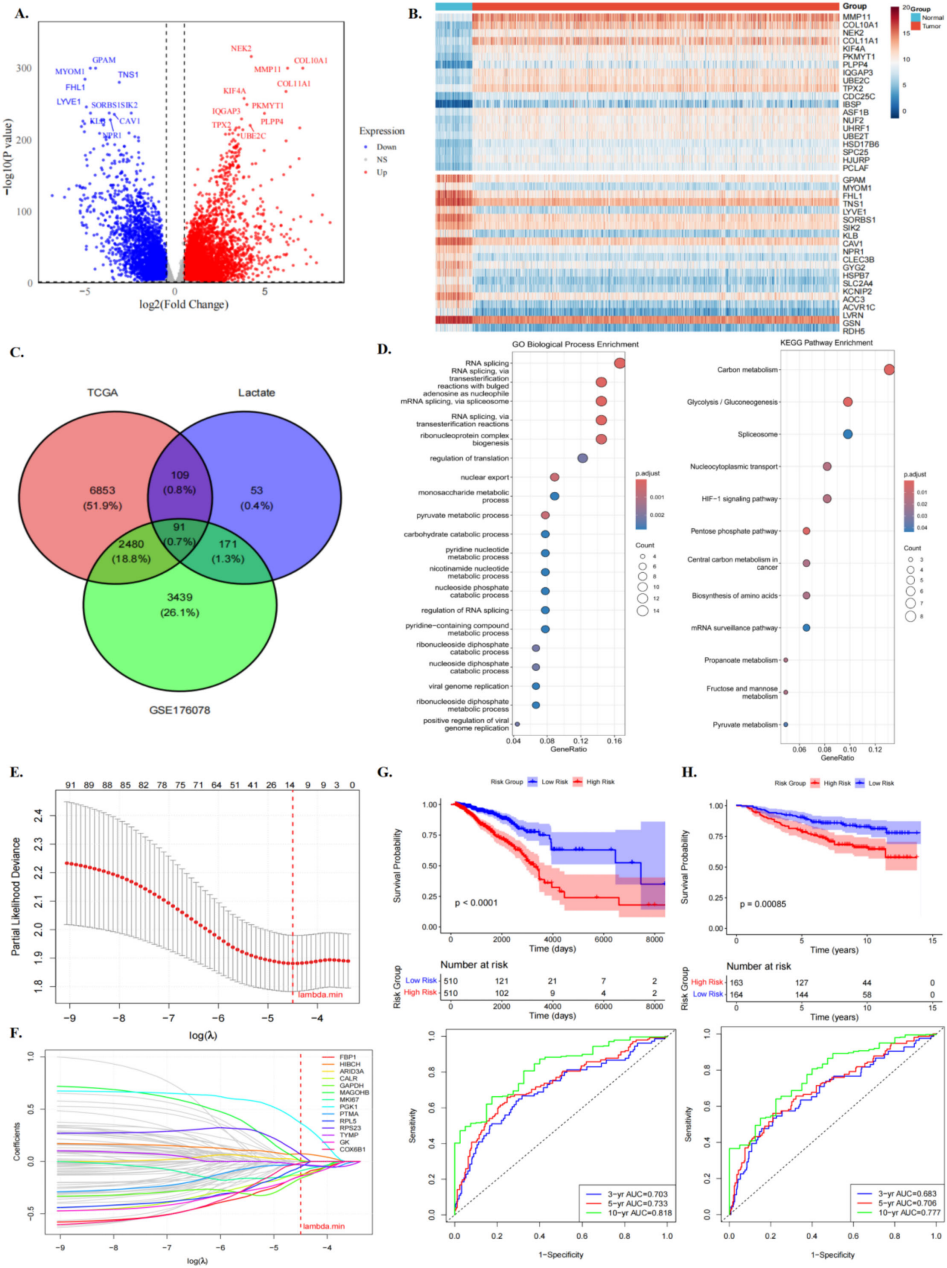
Construction of prognostic model. **(A)** TCGA-BRCA difference analysis volcano diagram. **(B)** Heat maps of the top 40 differentially expressed genes. **(C)** Venn analysis can identify overlapping genes among lactate activity characteristics, TCGA-BRCA and GSE176078 differential expression profiles. **(D)** GO and KEGG pathway analysis of differentially expressed genes. **(E)** Partial-likelihood bias varying with log(λ). **(F)** Visualisation of the lasso regression lasso coefficient trajectory. **(G)** Survival curves and time-varying ROC curves for the training set (TCGA-BRCA). **(H)** Survival curve and time-varying ROC curve for the validation set (GSE20685).

We first performed differential expression analysis between tumour and adjacent standard samples in the TCGA-BRCA dataset, identifying 5,957 upregulated and 3,576 downregulated genes, and visualised the expression dynamics of the top 40 genes using heatmaps (**Figures 3A, B**). To prioritize candidate genes for prognostic model construction, we integrated three complementary sources of evidence: (i) genes differentially expressed between high- and low-lactylation-activity epithelial cells at the single-cell level, (ii) the literature-curated lactylation-related reference gene pool, and (iii) tumour-associated genes identified from bulk tumour-versus-normal comparison in TCGA-BRCA. We emphasize that the bulk TCGA comparison was used as a supportive prioritization filter to enrich for breast cancer-relevant genes, rather than as a direct representation of lactylation biology. Intersecting these gene sets yielded 91 candidate genes for downstream prognostic screening(**Figure 3C**). GO and KEGG enrichment analyses revealed that these genes were primarily involved in RNA splicing and post-transcriptional regulatory processes, as well as energy metabolism-related pathways, including glycolysis, pyruvate metabolism, and the HIF-1 signalling pathway (**Figure 3D**). To identify core prognostic genes, LASSO regression was applied to the 91 candidate genes, resulting in 14 key genes and their corresponding coefficients (**Figures 3E, F**). To further explore the potential functions of these candidate genes and their roles in biological processes, a protein–protein interaction (PPI) network was constructed, and the expression levels of these genes were compared between tumour and standard samples. The results indicated that, except for RPL5, RPS23, and HIBCH, which were expressed at lower levels than in normal tissues, all other genes were significantly upregulated in tumours (P < 0.001) (**Supplementary Figures 1E, F**). Patients were then stratified into high- and low-risk groups using the median risk score as the threshold. Kaplan–Meier survival analysis revealed a significant prognostic difference between high- and low-risk groups (P < 0.001), with consistent results across both the TCGA training and external validation cohorts. Furthermore, time-dependent ROC curve analysis confirmed the predictive performance of the risk model (**Figures 3G, H**; **Supplementary Figures 4C, D**). Repeated 10-fold cross-validation and bootstrap validation further indicated that the model had modest but stable prognostic performance, although some degree of optimism was observed (**Supplementary Figures 4E, F**).

### 3.4 Clinical feature analysis and nomogram construction

**Figure 4:**
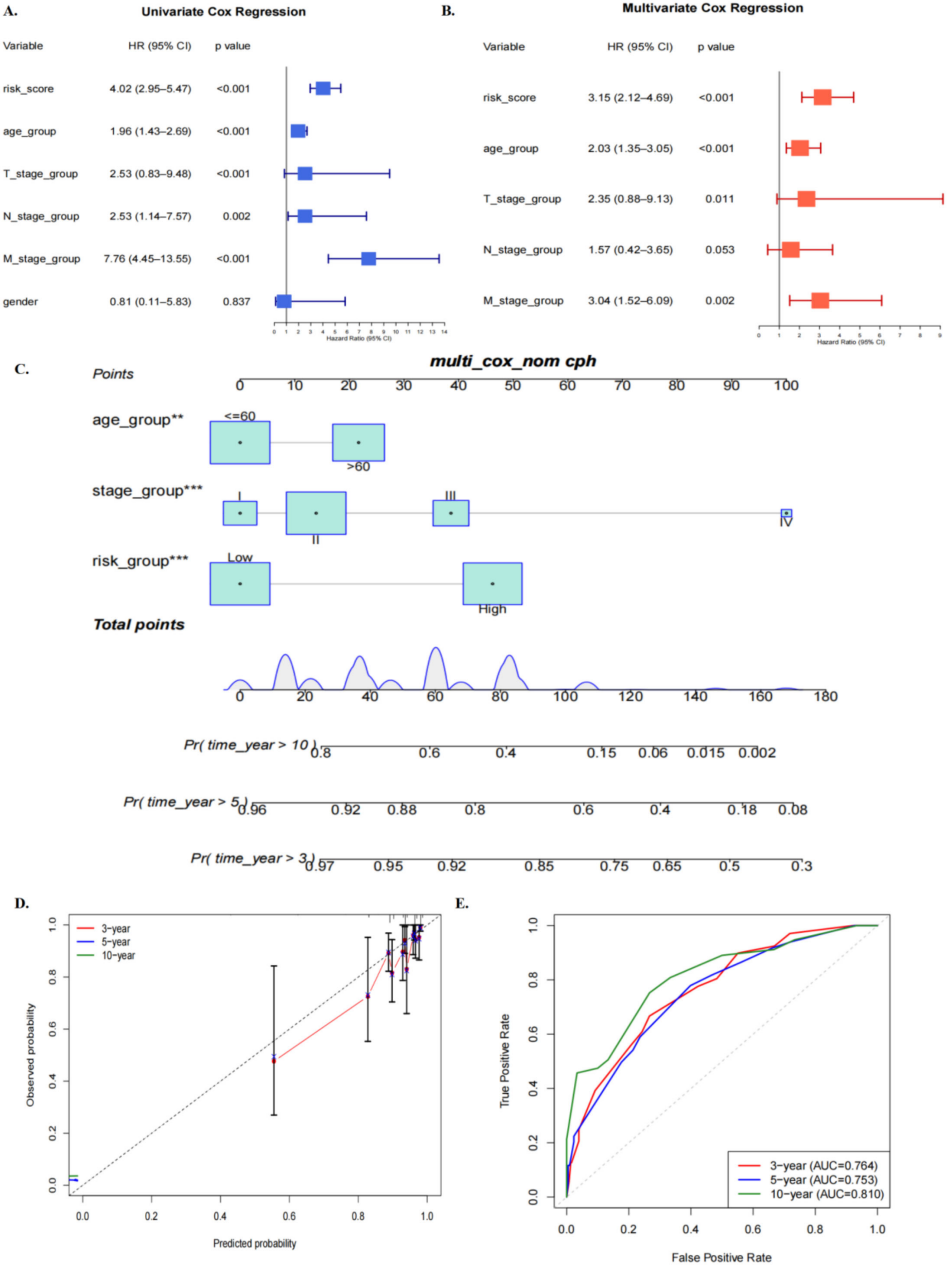
Independent prognostic factor analysis and nomogram construction. **(A)** Results of univariate cox regression analysis of risk score and clinical characteristics. Multivariate cox regression analysis results of the **(B)** risk score and clinical characteristics. **(C)** A nomogram for predicting OS in breast cancer patients. **(D)** Calibration curve for evaluating the accuracy of OS prediction. **(E)** ROC curves of the nomogram for predicting 3, 5, and 10-year survival.

We evaluated correlations between variables, including risk score, age group, T stage, N stage, M stage, and gender, and patient survival. In the univariate Cox regression analysis, the risk score was the strongest prognostic predictor (HR = 4.02, 95% CI: 2.95-5.47, P < 0.001); Age, T stage, N stage and M stage all significantly increased the risk of survival, while gender (HR ≈ 0.81, P = 0.837) had no significant effect on prognosis (**Figure 4A**). In the multivariate Cox analysis, the adjusted risk score, age, T stage, and M stage remained independent prognostic factors, while the influence of N stage was attenuated. This indicates that, compared with traditional clinical features, the risk score has greater independence and predictive ability for prognosis (**Figure 4B**). Based on these results, we constructed a nomogram integrating clinical variables and risk scores to predict survival probabilities at 3, 5, and 10 years (**Figure 4C**). The calibration curve shows that the predicted values at each time point are highly consistent with the actual observed values, and the confidence intervals are close to the ideal diagonal (**Figure 4D**). Time-dependent ROC curve analysis further confirmed the model’s discriminative power, with AUCs of 0.764, 0.753, and 0.810 at 3, 5, and 10 years, respectively (**Figure 4E**). Decision curve analysis further showed that the nomogram provided a higher net benefit than the treat-all and treat-none strategies across a broad range of threshold probabilities, supporting its potential clinical utility(**Supplementary Figure 5A**). Together, these findings suggest that the combined model may offer incremental prognostic information beyond conventional clinicopathological variables, although its clinical utility will require further validation in additional independent cohorts.

### 3.5 Mutation landscape, immune infiltration and drug sensitivity under lactylation risk stratification

Immune infiltration analysis revealed group differences in several inferred immune/stromal features, and MCP-counter broadly supported the major trends identified by CIBERSORT, particularly reduced lymphocyte-associated signals and relatively increased fibroblast-related signals in the high-risk group. Correlation analysis further suggested that several prognostic genes were associated with specific immune cell populations. In contrast, statistical comparison of mutation frequencies did not identify robust group differences after FDR correction, and tumour mutation burden was also not significantly different between the two groups. The oncoPredict-based drug sensitivity analysis suggested exploratory differences in predicted response between risk groups; however, these findings should be interpreted as hypothesis-generating only and not as direct evidence of treatment efficacy or subtype-specific clinical utility.

**Figure 5:**
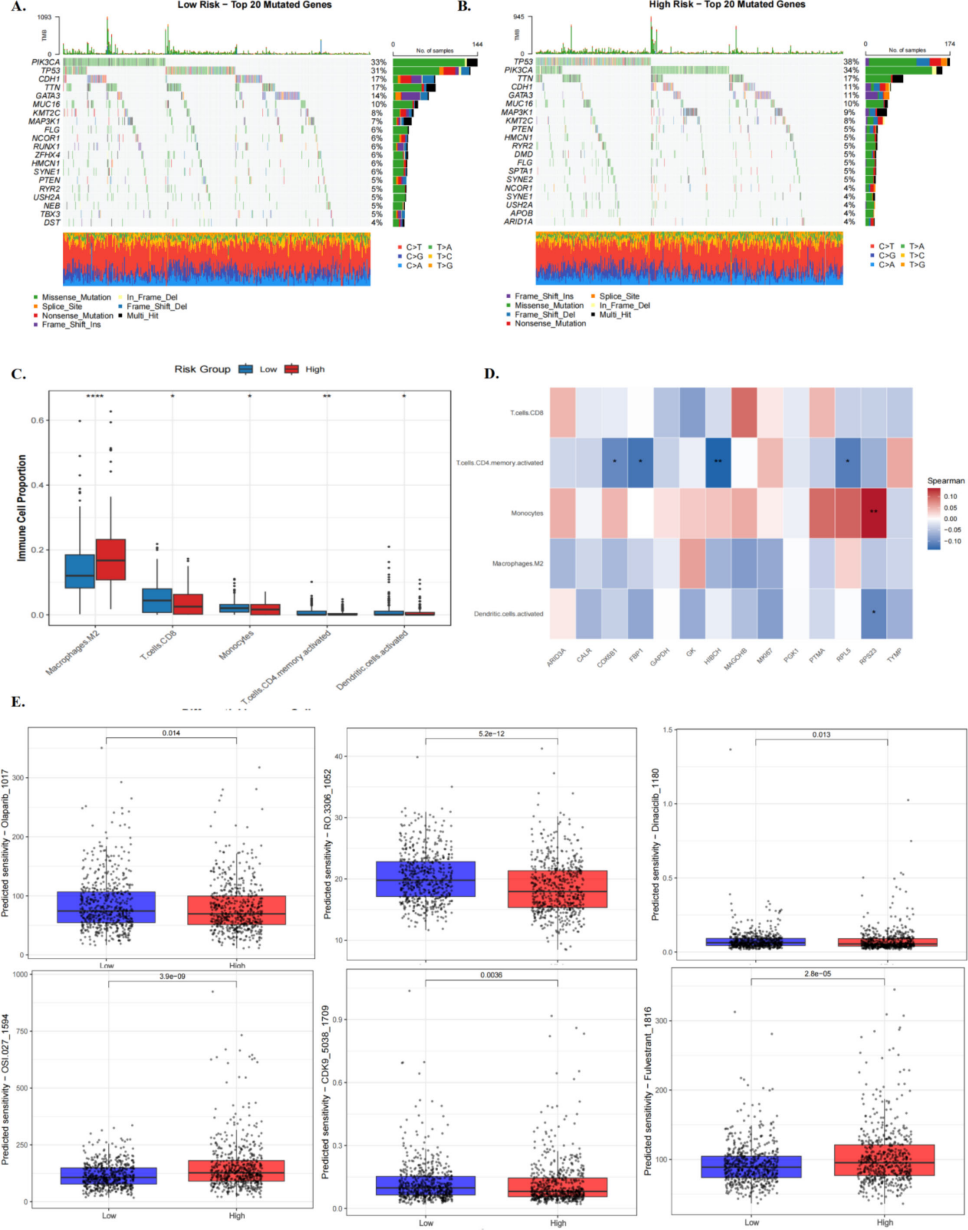
Analysis of mutation landscape, immune microenvironment and drug sensitivity in high and low-risk groups. **(A)** Landscape map of the Top 20 mutant genes in the low-risk group. **(B)** A similar landscape map of the Top 20 mutant genes in the high-risk group. **(C)** Box plot of differences in immune cell infiltration. **(D)** Correlation analysis between differential immune cells and prognostic genes. **(E)** Box plot of drug sensitivity. * indicates p < 0.05, ** indicates p < 0.01.

We systematically analysed TCGA-BRCA mutation data using the R package maftools. In the low-risk group, common mutated genes included PIK3CA, GATA3, MAP2K1, ZFXR1, and TP53, predominantly harbouring missense mutations, with relatively few splice-site mutations or in-frame deletions, suggesting that the low-risk group’s genome was relatively stable. Approximately 33% of low-risk patients harboured PIK3CA mutations (**Figure 5A**). In contrast, the high-risk group was primarily characterised by TP53 mutations (38%) and exhibited a modest increase in stop codon mutations, reflecting higher genomic instability and providing a rationale for exploring associations between mutation patterns and disease risk (**Figure 5B**). Statistical comparison of mutation frequencies did not identify robust differences between risk groups after FDR correction. Similarly, TMB was not significantly different between the two groups (Wilcoxon P = 0.871)(**Supplementary Figures 5B, C**). Immune infiltration analysis revealed significant differences in five of 22 immune cell types between the high- and low-risk groups. Notably, M2 macrophage infiltration was significantly elevated in the high-risk group (**Figure 5C**). MCP-counter broadly supported the major immune/stromal trends identified by CIBERSORT, particularly reduced lymphocyte-associated signals and relatively increased fibroblast-related signals in the high-risk group, although complete concordance across all inferred cell subsets was not expected (**Supplementary Figure 5D**). Further correlation analysis indicated that some prognostic genes were significantly associated with immune cell populations: HIBCH exhibited the strongest negative correlation with activated CD4 memory T cells, whereas RPS23 showed the strongest positive correlation with monocytes (**Figure 5D**). These findings indicate marked differences in the immune microenvironment between risk groups and suggest that the identified prognostic genes could serve as potential targets for personalised breast cancer therapy. Drug sensitivity prediction demonstrated that most targeted agents, including the PARP inhibitor Olaparib (P = 0.014; median IC50 decreased by ~20%), CDK inhibitors Dinaciclib (P = 0.013) and RO-3306 (P = 5.16e-12), as well as CDK9 inhibitors (P = 0.004), exhibited higher sensitivity in the low-risk group. In contrast, the mTOR inhibitor OSI-027 (P = 3.86e-09) and the endocrine therapy agent Fulvestrant (P = 2.84e-05) were more effective in the high-risk group.

### 3.6 CALR knockdown suppresses the proliferative capacity of breast cancer cells *in vitro*

**Figure 6:**
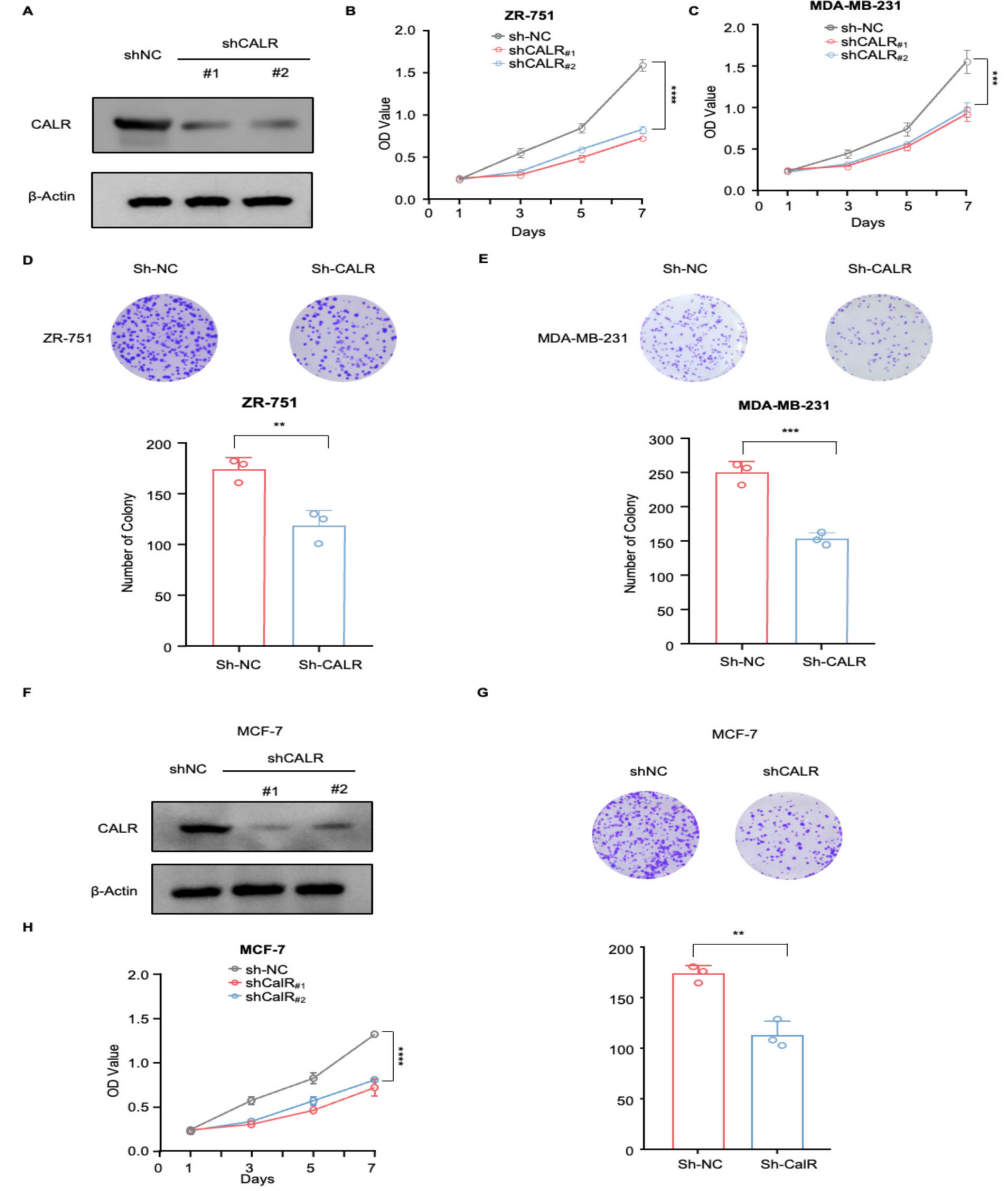
Knockdown of CALR inhibits the proliferation of breast cancer cells *in vitro*. **(A, F)** Western blot analysis confirming the efficiency of CALR knockdown by two independent siRNAs (shCALR#1 and shCALR#2) in ZR-75-1, MCF-7 and MDA-MB-231 cells, with β-Actin as a loading control. **(B, C, H)** Cell proliferation was determined by CCK-8 assays at the indicated time points. ZR-75-1 **(B)**, MDA-MB-231 **(C)**, and MCF-7 **(H)** cells with CALR knockdown exhibited significantly lower OD values than shNC controls. **(D, E, G)** Representative images and quantitative analysis of colony formation assays showing that CALR knockdown markedly reduced colony number and size in ZR-75-1 **(D)**, MDA-MB-231 **(E)**, and MCF-7 **(G)** cells. Statistical significance: *P < 0.05; **P < 0.01; ***P < 0.001; ****P < 0.0001; Student’s t-test.

To validate computational predictions, CALR expression was suppressed by two independent siRNAs.Western blot analysis confirmed effective knockdown in ZR-751, MCF-7 and MDA-MB-231 cells (**Figure 6A**). CCK-8 assays demonstrated that CALR depletion significantly inhibited cell proliferation over seven days (**Figures 6B, C**), and colony-formation assays revealed marked reductions in colony number and size compared with controls (**Figures 6D, E**). These results indicate that CALR is essential for maintaining the proliferative capacity of breast cancer cells.

**Figure 7:**
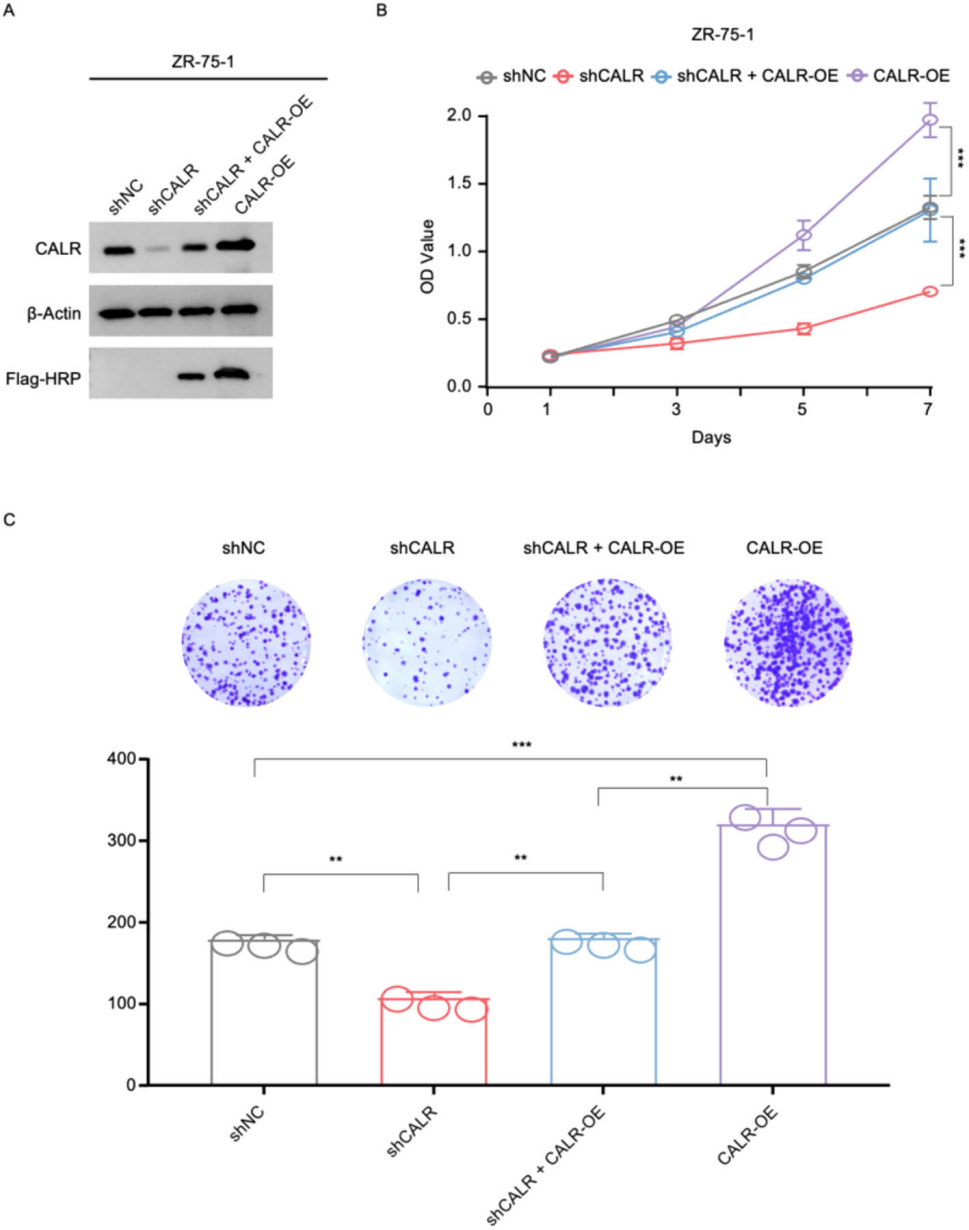
Rescue experiments confirm the role of CALR in regulating breast cancer cell proliferation. **(A)** Western blot analysis showing CALR expression in ZR-75–1 cells under shNC, shCALR, shCALR + CALR-OE, and CALR-OE conditions. β-Actin was used as a loading control, and Flag-HRP confirmed exogenous CALR expression. **(B)** Cell proliferation was assessed by CCK-8 assays. CALR knockdown significantly reduced proliferation, which was partially restored by CALR re-expression, while CALR overexpression alone further enhanced cell growth. **(C)** Representative images and quantitative analysis of colony formation assays showing reduced clonogenic capacity following CALR knockdown, partial rescue upon CALR re-expression, and increased colony formation in CALR-overexpressing cells. Statistical significance: *P < 0.05; **P < 0.01; ***P < 0.001; Student’s t-test.

To further evaluate the functional role of CALR and minimise the potential influence of off-target effects, rescue experiments were performed in ZR-75–1 cells. Western blot analysis confirmed effective CALR knockdown and re-expression following CALR overexpression (**Figure 7A**). CCK-8 assays showed that CALR depletion was associated with reduced cell proliferation, whereas re-expression of CALR partially restored proliferative capacity. In contrast, CALR overexpression alone was associated with enhanced cell growth compared with control cells (**Figure 7B**). Consistently, colony formation assays showed that CALR knockdown reduced colony numbers, while CALR re-expression partially reversed this effect. CALR overexpression was associated with increased clonogenic capacity (**Figure 7C**). Together, these findings support a role for CALR in breast cancer cell proliferation and suggest that its effects are at least partially reversible, although the precise underlying mechanisms remain to be further elucidated.

### 3.7 CALR depletion induces G1-phase cell-cycle arrest and promotes apoptotic cell death

**Figure 8:**
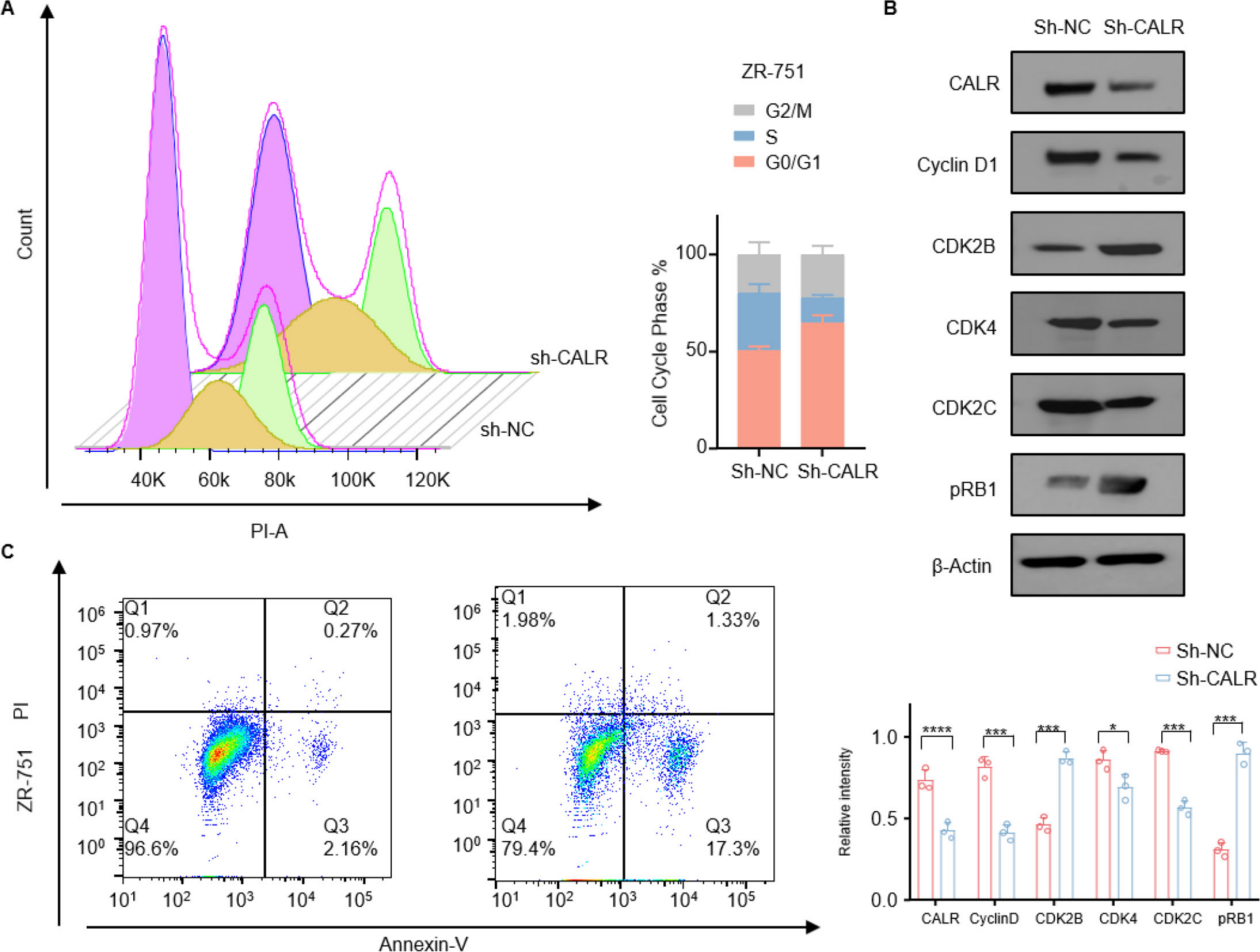
CALR depletion induces G1-phase arrest and apoptosis. **(A)** Flow-cytometric analysis of cell-cycle distribution in ZR-751 cells following CALR knockdown. Compared with the shNC control, the shCALR group exhibited a pronounced increase in the G0/G1 population and a corresponding decrease in S-phase cells, indicating G1-phase arrest. **(B)** Western blot analysis showing that CALR knockdown downregulated key cell-cycle regulators, including Cyclin D1, CDK2B, CDK4, and CDK2C, and decreased RB1 phosphorylation (pRB1). β-Actin served as the loading control. The right panel presents quantitative densitometric analysis of relative protein expression levels. **(C)** Annexin V/PI staining and flow-cytometric detection of apoptosis in ZR-751 cells. CALR knockdown markedly increased the proportion of early- and late-apoptotic cells compared with the shNC control. Quantitative data are shown as mean ± SD (n = 3).

Flow-cytometric analysis showed that CALR-silenced ZR-751 cells accumulated in G_0_/G_1_ phase with a corresponding decrease in S-phase population, confirming G1-phase arrest (**Figure 8A**). Western-blot analysis revealed down-regulation of key G1/S regulators—Cyclin D1, CDK4, CDK2B, CDK2C—and decreased phosphorylation of RB1 (**Figure 8B**). Consistent with impaired cell-cycle progression, Annexin V/PI staining demonstrated a significant increase in both early and late apoptosis among CALR-depleted cells relative to controls (**Figure 8C**). These findings suggest that CALR may be involved in regulating cell cycle progression and survival in breast cancer cells, although further studies are required to elucidate the precise molecular mechanisms.However, these findings primarily indicate a functional association, and the precise upstream mechanisms linking the lactylation–CALR axis to cell cycle regulation remain to be further elucidated.

### 3.8 CalR-knockdown suppresses tumour growth and proliferative activity *in vivo*

**Figure 9:**
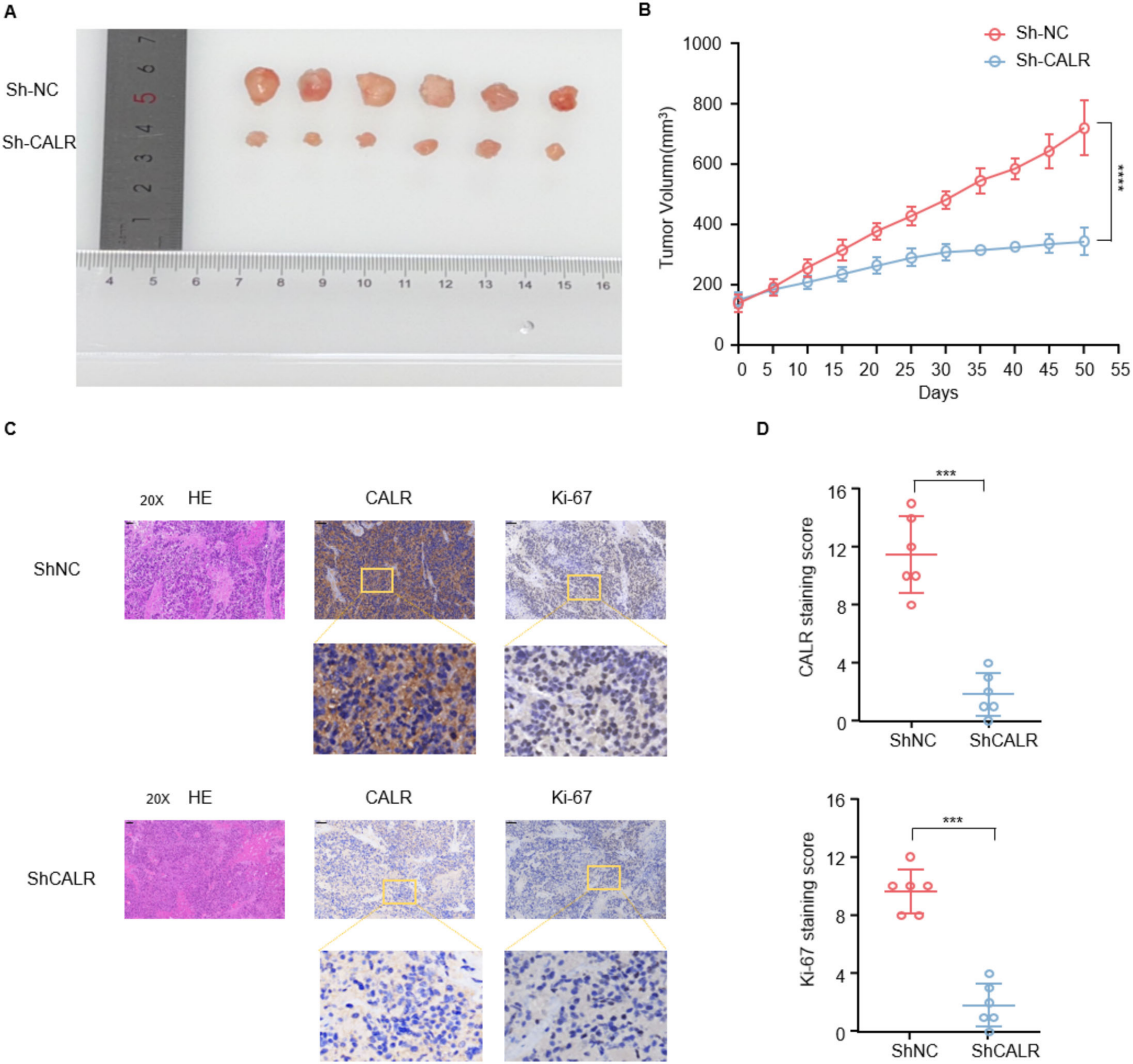
CALR -knockdown suppresses tumour growth and proliferation *in vivo*. **(A)** Representative images of xenograft tumours derived from ZR-751 cells stably expressing shNC or shCALR. Tumours in the shCALR group were markedly smaller than those in the shNC control group. **(B)** Tumour growth curves showed that CALR knockdown significantly inhibited tumour growth throughout the observation period. Tumour volume was measured every five days, and data are presented as mean ± SD (n=6). **(C)** Representative haematoxylin–eosin(H&E) and immunohistochemical (IHC) staining for CALR and Ki-67 in xenograft tumour tissues. The shCALR group showed markedly reduced CALR expression and a lower Ki-67 proliferation index than the shNC group. Scale bars: 50 μm (upper panels). **(D)** Quantitative analysis of CALR and Ki-67 staining scores showing significant decreases in CALR expression and proliferative activity in the shCALR group. Data are expressed as mean ± SD (n = 6).

The tumour-suppressive role of CALR was further confirmed *in vivo* using ZR-751 cell-derived xenografts. Mice implanted with CALR-knockdown cells developed significantly smaller tumours than controls (**Figures 9A, B**). H&E staining revealed reduced cellularity and looser tissue architecture in the shCALR group. Immunohistochemistry confirmed diminished CALR and Ki-67 expression, with quantitative scoring showing significant reductions in both markers (**Figures 9C, D**). These results support the notion that CALR is associated with tumour growth in breast cancer. However, given that the *in vivo* experiments were performed in a single cell line model, further validation in additional subtypes will be necessary to confirm the generalisability of these findings.

## 4 Discussion

This study provides a comprehensive single-cell transcriptomic landscape of the breast cancer TME and establishes lactylation as a pivotal metabolic mechanism that coordinates immune and stromal remodelling. By integrating 26 scRNA-seq datasets covering ER+, HER2+, and TNBC subtypes, we resolved 47 transcriptionally distinct clusters across six major cell lineages. The predominance of epithelial and T-cell populations, together with subtype-specific variations—fewer endothelial cells in TNBC and fewer epithelial cells in HER2+ tumours—underscores the metabolic and immune diversity that shapes clinical behaviour (36, 37).

A key finding of our study is the predominant enrichment of lactylation-related activity within epithelial cells, with the TNBC subtype exhibiting the highest intensity. This observation implicates lactylation as a driver of subtype-specific metabolic reprogramming (38). Lactylation-a lactate-derived post-translational modification—serves as a biochemical bridge between glycolytic flux and transcriptional regulation. Consistent with this role, our single-cell analyses revealed that highly lactylated epithelial and fibroblast clusters exhibited enhanced communication with the myeloid and T-cell compartments, establishing an immunosuppressive metabolic niche (39). GO and KEGG enrichment indicated activation of immune response, cell cycle, and hypoxia-related pathways, mirroring the HIF-1α-driven adaptation seen in glycolysis-dominant tumours. These findings suggest that excessive lactylation reinforces metabolic plasticity and promotes immune evasion—mechanisms particularly relevant to TNBC’s aggressiveness.

By translating these molecular insights into clinical relevance, we developed a 14-gene lactylation-associated prognostic model derived from the TCGA and GSE20685 cohorts (40). This signature achieved robust prognostic stratification (HR = 4.02, P < 0.001) and outperformed conventional clinicopathological predictors. The model effectively captured the intersection of metabolic and transcriptional dysregulation, revealing that TP53 mutations and M2 macrophage infiltration characterised high-risk tumours. In contrast, low-risk tumours exhibited PIK3CA and GATA3 mutations (38), indicative of genomic stability and endocrine responsiveness. Drug-sensitivity analysis further suggested that lactylation status may predict therapeutic vulnerability: high-risk tumours showed greater susceptibility to mTOR and endocrine therapies, whereas low-risk tumours were more sensitive to PARP and CDK inhibitors. These results highlight the potential of lactylation-based risk stratification to guide precision treatment.

CALR was selected for functional validation based on a combination of factors, including its inclusion in the 14-gene prognostic signature, its stable association with adverse prognosis/risk stratification, its biological relevance to endoplasmic reticulum stress and calcium homeostasis, and its experimental tractability in breast cancer models. Experimental validation provided additional support for the computational findings. CALR, an endoplasmic reticulum chaperone and calcium-binding protein implicated in protein folding and immune regulation, was selected for functional validation based on its contribution to the prognostic model, consistent expression patterns across datasets, and its reported involvement in tumour-associated biological processes (41, 42). Functional assays demonstrated that CALR knockdown markedly suppressed proliferation (43), induced G1-phase arrest, and promoted apoptosis in breast cancer cells, thereby phenocopying the computationally predicted anti-proliferative effects of lactylation inhibition.

For mechanistic investigations, we primarily focused on the ZR-75–1 cell line, a hormone receptor–positive model, due to its stable and reproducible responses following CALR knockdown, as well as the well-characterised Cyclin D–CDK–RB1 regulatory axis in this subtype. Within this model, CALR depletion was associated with reduced expression of key G1/S transition regulators, including Cyclin D1 and CDK4, as well as decreased RB1 phosphorylation, suggesting a potential association in cell cycle regulation. However, these findings primarily reflect a functional association rather than a defined mechanistic pathway, and the precise upstream regulatory mechanisms linking the lactylation–CALR axis to these cell cycle regulators remain to be elucidated. Notably, the partial rescue of proliferative capacity upon CALR re-expression is consistent with a potential functional association between CALR and cell proliferation, while not establishing a direct mechanistic pathway. *In vivo* experiments using a ZR-75–1 xenograft model further supported the *in vitro* observations, as CALR knockdown led to reduced tumour growth and decreased Ki-67 expression. While these findings indicate that CALR may be associated with tumour proliferative capacity, it should be noted that the use of a single cell line model may limit the generalisability of these results. Future studies incorporating additional breast cancer subtypes, particularly metabolically distinct TNBC models, will be necessary to validate these observations. The xenograft experiment should be interpreted as a proof-of-concept validation rather than as a subtype-spanning *in vivo* representation of the full single-cell lactylation score. We selected a hormone receptor-positive breast cancer cell line as a tractable *in vivo* model, but this choice does not imply that the observed biology is equally representative of triple-negative breast cancer. Importantly, the lactylation score used in the single-cell analysis reflects a literature-curated lactylation-associated transcriptional state rather than direct quantification of protein lactylation itself. Therefore, the xenograft data are best interpreted as supporting the biological relevance of the identified high-risk/lactylation-associated features in one breast cancer context, while additional validation in TNBC-oriented systems will be needed in future work.

Several recent studies have already proposed lactylation-related prognostic models in breast cancer, including cluster-based, metastasis-oriented, and machine-learning-derived signatures linked to prognosis, immune context, and therapeutic prediction. Compared with these studies, the present work attempts to connect a bulk lactylation-associated prognostic model with single-cell-resolved transcriptional states and microenvironmental interpretation. At the same time, our findings should be interpreted cautiously because the current score remains transcript-based and does not directly quantify lactylation modification itself. Thus, the present study is better viewed as identifying lactylation-associated transcriptional patterns than as establishing definitive lactylation-driven mechanisms (29, 44, 45).

The therapeutic implications of lactylation should also be framed more concretely and cautiously. Rather than implying immediate clinical applicability, our findings support testable hypotheses in tumours with high-risk features linked to glycolytic and stromal remodelling. One possible strategy is to target upstream lactate production or transport, such as LDHA- or MCT-related pathways, to reduce lactate availability for lactylation-associated signalling. A second possibility is to modulate candidate writer/eraser systems, including p300- and HDAC/SIRT-related pathways, to perturb lactylation-associated chromatin or protein states. However, these therapeutic inferences remain speculative and require direct mechanistic and pharmacologic validation (46, 47).

Collectively, our study positions lactylation as a dynamic metabolic rheostat within the breast cancer TME, regulating both epithelial plasticity and immune evasion (48). By coupling single-cell transcriptomics with *in vitro* and *in vivo* validation, we provide compelling evidence that lactylation not only remodels the metabolic landscape but also sustains an immunosuppressive milieu favourable for tumour progression. Clinically, the lactylation-derived prognostic model may serve as a precision tool for risk stratification and treatment optimisation. Integrating this score into adaptive clinical frameworks—for example, combining lactate dehydrogenase blockade or bromodomain inhibitors with CDK4/6 inhibitors in high-risk TNBC—could unveil synergistic therapeutic avenues. Moreover, lactylation profiling may identify patients more likely to benefit from immunometabolic interventions.

Several limitations should be acknowledged. First, the lactylation score used in this study is an indirect measure derived from gene-expression patterns rather than a direct quantification of protein lactylation modification. It should therefore be interpreted as a transcript-level surrogate of lactylation-associated biology rather than a direct readout of lactylation status. Second, the present study is fundamentally correlative and does not establish causality between lactylation and the observed clinical, immune, or microenvironmental associations. Third, the tumour-versus-normal comparison in TCGA-BRCA should be interpreted cautiously because the normal samples are limited and largely unpaired, and the observed expression differences may also be influenced by subtype composition and tumour purity. Fourth, although additional validation was performed in GSE20685 and METABRIC, the prognostic model showed only modest discrimination in internal resampling, with measurable optimism in the training cohort. Fifth, the oncoPredict results are exploratory and hypothesis-generating only, and should not be interpreted as clinically actionable predictions. Finally, we did not perform direct validation in patient tissue samples to confirm the relationships among the prognostic model, lactylation levels, and clinicopathological characteristics. Future studies integrating direct lactylation measurements, spatial transcriptomics, and prospective multi-omics cohorts will be required to strengthen mechanistic and clinical inference.

In summary, this integrative analysis defines lactylation as a central metabolic mechanism underpinning breast cancer heterogeneity and progression. By elucidating its single-cell architecture and validating key functional mediators, we establish lactylation as both a mechanistic hallmark and a clinically actionable metabolic vulnerability. These insights lay the foundation for metabolism-directed therapeutic strategies that transcend conventional subtype classification and advance precision oncology in breast cancer.

## 5 Conclusion

This integrative single-cell and experimental study delineates lactylation as a central metabolic mechanism that remodels the breast cancer microenvironment. The 14-gene lactylation-associated signature provides a robust prognostic tool. It identifies lactylation as a therapeutically targetable metabolic axis, offering new opportunities for precision stratification and metabolism-guided intervention in breast cancer.

## Data Availability

The original contributions presented in the study are included in the article/**Supplementary Material**. Further inquiries can be directed to the corresponding authors.

## Supplementary material

Supplementary Figure 1 Single-cell transcriptome subtype analysis and PPI network construction of breast cancer. **(A)** The UMAP dimensionality reduction map shows the clustering of cell clusters. **(B)** Stratified cell type statistics by molecular subtypes (ER+, HER2+ and TNBC). **(C)** UMAP view of lactic acid activity stratified by molecular subtypes (ER+, HER2+, and TNBC), highlighting the lactic acid expression of subtypes. **(D)** The expression of lactic acid activity stratified by molecular subtypes (ER+, HER2+ and TNBC) in each cell type. **(E)** PPI network diagram of prognostic genes. **(F)** Box plot of expression differences of prognostic genes in tumours and normal tissues.

Supplementary Figure 2 Sensitivity analysis using the external 22-gene lactylation-related panel in breast cancer. **(A)** Violin/box plots showing the distribution of the external 22-gene lactylation-associated module score across major cell types. **(B)** Heatmap showing the relative score patterns across major cell types stratified by molecular subtypes (ER+, HER2+, and TNBC). **(C)** Gene Ontology (GO) enrichment analysis of differentially expressed genes between the high-score and low-score states defined by the external 22-gene panel. **(D)** KEGG pathway enrichment analysis of differentially expressed genes between the high-score and low-score states defined by the external 22-gene panel.

Supplementary Figure 3 Sensitivity analysis under alternative cutoff strategies for the lactylation-associated module score. **(A)** Density distribution of the module score showing the positions of median, tertile, and quartile cutoffs. **(B)** Numbers of significantly upregulated and downregulated genes across major cell types under tertile- and quartile-based grouping strategies. **(C)** GO enrichment analysis of differentially expressed genes identified under tertile- and quartile-based cutoff strategies. **(D)** KEGG pathway enrichment analysis of differentially expressed genes identified under tertile- and quartile-based cutoff strategies.

Supplementary Figure 4 Additional immune-signature analyses and supplementary validation of prognostic model performance. **(A)** Comparison of T-cell exhaustion, Treg, and myeloid M2-like module scores between the low-risk and high-risk groups. **(B)** Heatmap summarising representative immune marker expression patterns across the two risk groups. **(C)** Time-dependent ROC curves for the 14-gene prognostic model in the METABRIC cohort. **(D)** Kaplan-Meier survival curves for the low-risk and high-risk groups in the METABRIC cohort. **(E)** Distribution of bootstrap optimism estimates for internal validation of the prognostic model. **(F)** Distribution of C-index values from repeated 10-fold cross-validation in the TCGA cohort.

Supplementary Figure 5 Additional clinical utility, genomic, and immune-stromal validation analyses of the prognostic model. **(A)** Decision curve analysis (DCA) of the nomogram model. **(B)** Differential mutation-frequency analysis between the high-risk and low-risk groups. Dot size indicates the magnitude of mutation-frequency difference, and colour indicates the group in which the mutation is enriched. **(C)** Comparison of tumour mutation burden (TMB) between the high-risk and low-risk groups. **(D)** MCP-counter analysis showing differences in estimated immune/stromal features between the low-risk and high-risk groups.
