# Supplementary material for: Lactylation-mediated remodelling of the breast cancer microenvironment: single-cell multidimensional analysis and prognostic model construction
Source: Front Immunol. 2026 May 13;17:1747043. doi: 10.3389/fimmu.2026.1747043 (PMC13212231; doi:10.3389/fimmu.2026.1747043)
Supplement: Supplementary file 1 [file Image1.pdf]

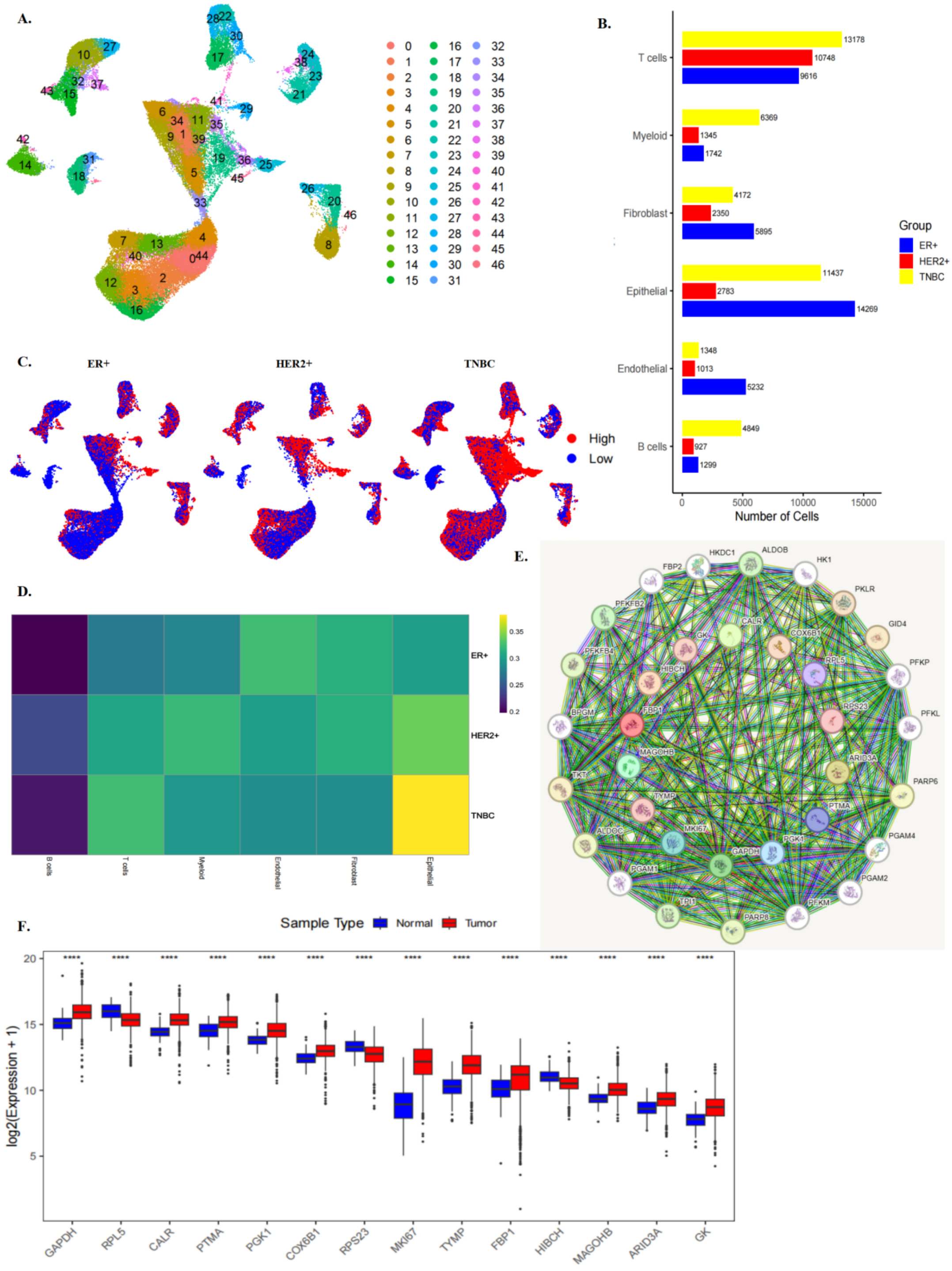

Supplementary Figure 1: Single-cell transcriptome subtype analysis and PPI network construction of breast cancer. (A) The UMAP dimensionality reduction map shows the clustering of cell clusters. (B) Stratified cell type statistics by molecular subtypes (ER+, HER2+ and TNBC). (C) UMAP view of lactic acid activity stratified by molecular subtypes (ER+, HER2+, and TNBC), highlighting the lactic acid expression of subtypes. (D) The expression of lactic acid activity stratified by molecular subtypes (ER+, HER2+ and TNBC) in each cell type. (E) PPI network

diagram of prognostic genes. (F) Box plot of expression differences of prognostic genes in tumours and normal tissues

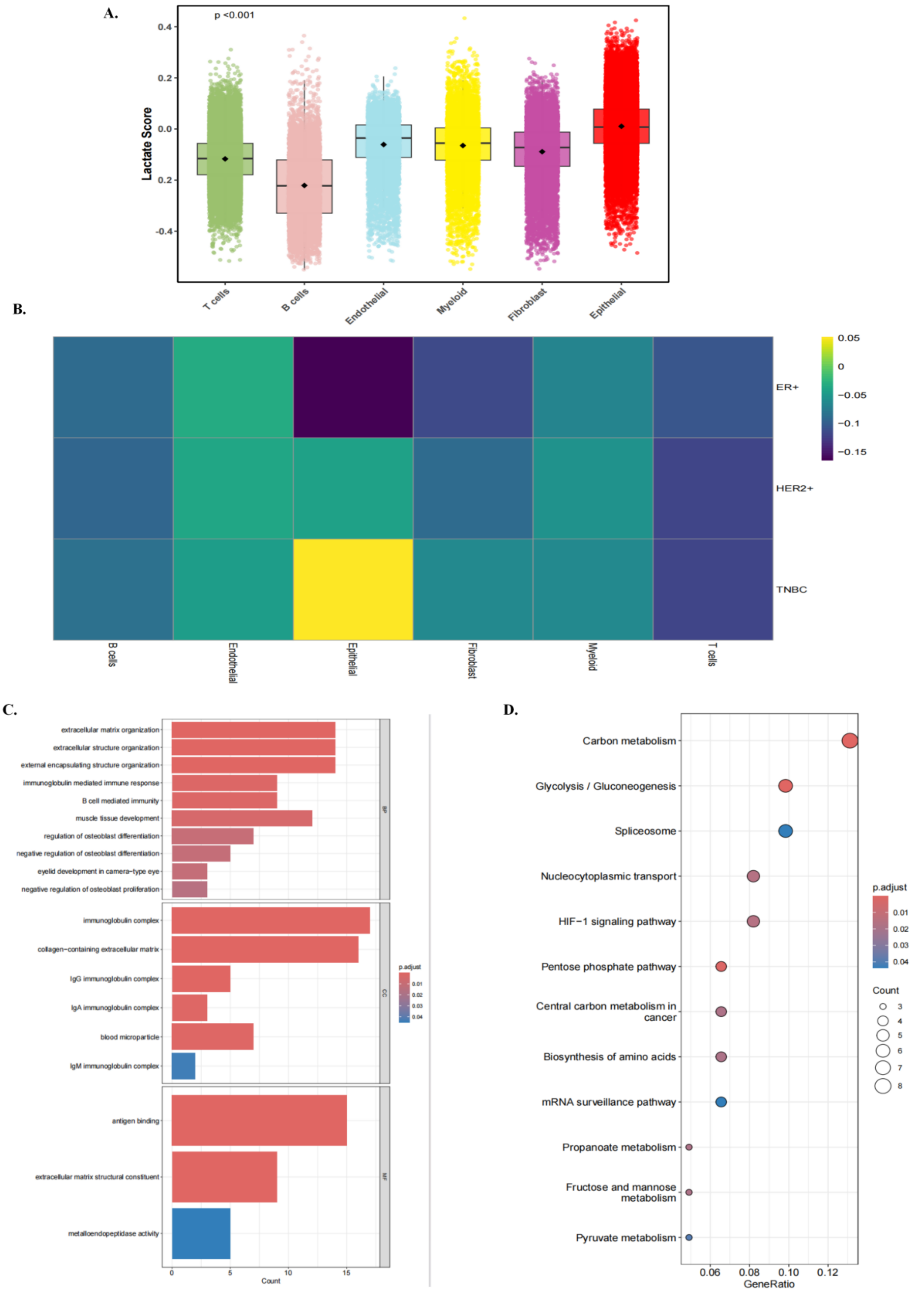

Supplementary Figure 2. Sensitivity analysis using the external 22-gene lactylation-related panel in breast cancer. (A) Violin/box plots showing the distribution of the external 22-gene lactylation-associated module score across major cell types. (B) Heatmap showing the relative score patterns

across major cell types stratified by molecular subtypes (ER+, HER2+, and TNBC). (C) Gene Ontology (GO) enrichment analysis of differentially expressed genes between the high-score and low-score states defined by the external 22-gene panel. (D) KEGG pathway enrichment analysis of differentially expressed genes between the high-score and low-score states defined by the external 22-gene panel.

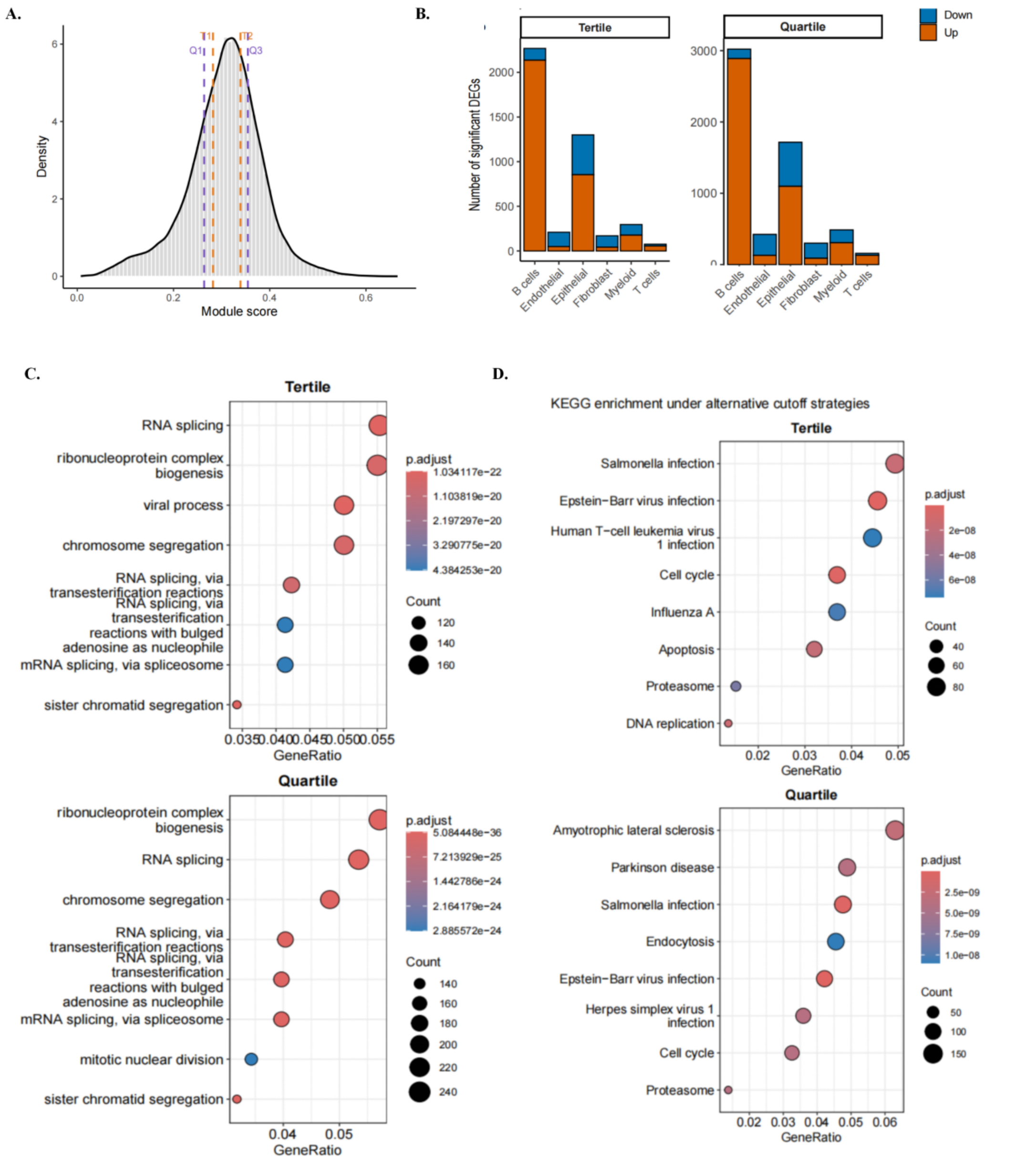

Supplementary Figure 3. Sensitivity analysis under alternative cutoff strategies for the lactylation-associated module score. (A) Density distribution of the module score showing the positions of median, tertile, and quartile cutoffs. (B) Numbers of significantly upregulated and downregulated genes across major cell types under tertile- and quartile-based grouping strategies. (C) GO enrichment analysis of differentially expressed genes identified under tertile- and quartile-based

cutoff strategies. (D) KEGG pathway enrichment analysis of differentially expressed genes identified under tertile- and quartile-based cutoff strategies

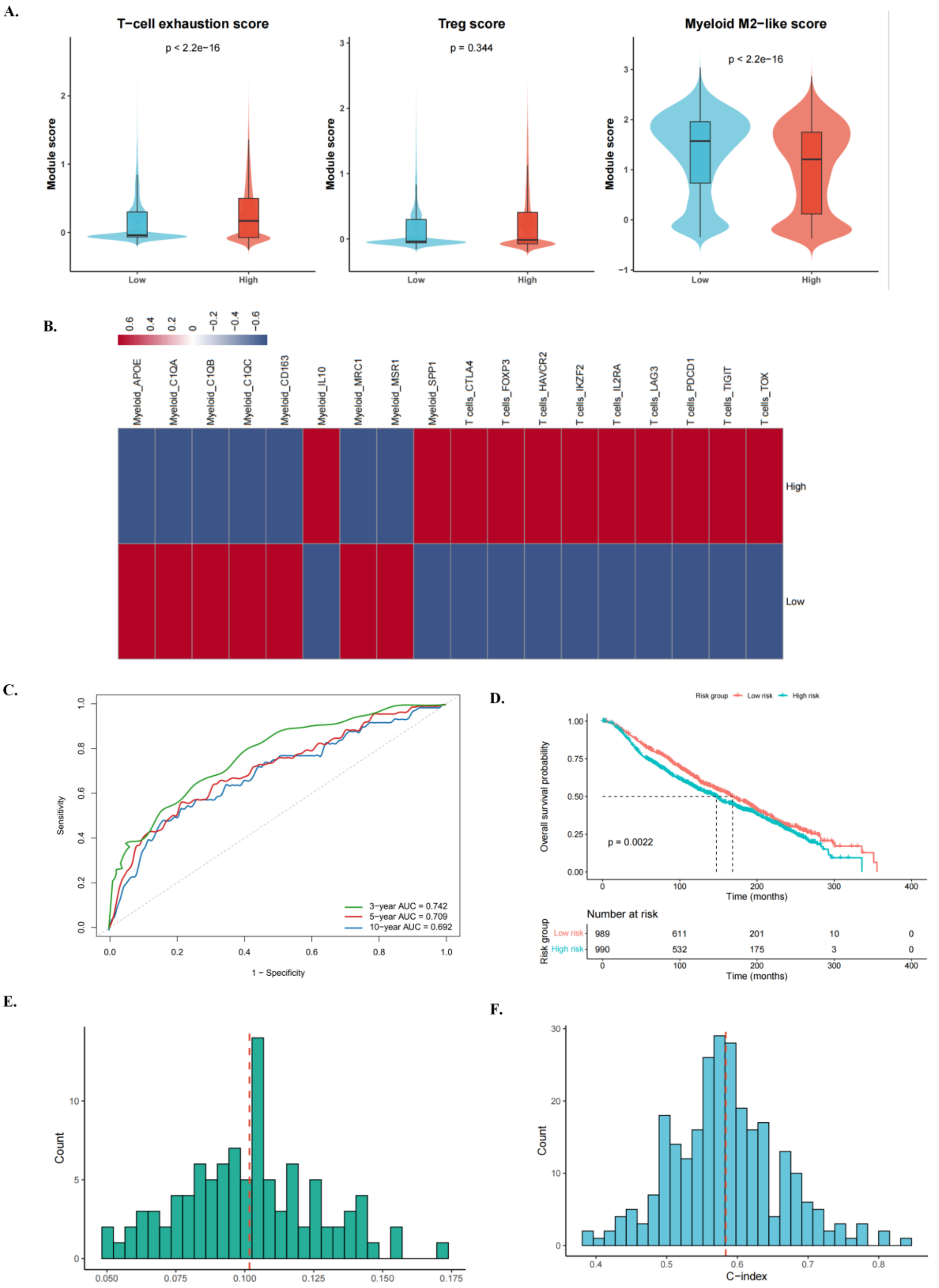

Supplementary Figure 4. Additional immune-signature analyses and supplementary validation of prognostic model performance. (A) Comparison of T-cell exhaustion, Treg, and myeloid M2-like module scores between the low-risk and high-risk groups. (B)Heatmap summarising

representative immune marker expression patterns across the two risk groups. (C) Time-dependent ROC curves for the 14-gene prognostic model in the METABRIC cohort. (D) Kaplan-Meier survival curves for the low-risk and high-risk groups in the METABRIC cohort. (E) Distribution of bootstrap optimism estimates for internal validation of the prognostic model. (F) Distribution of C-index values from repeated 10-fold cross-validation in the TCGA cohort.

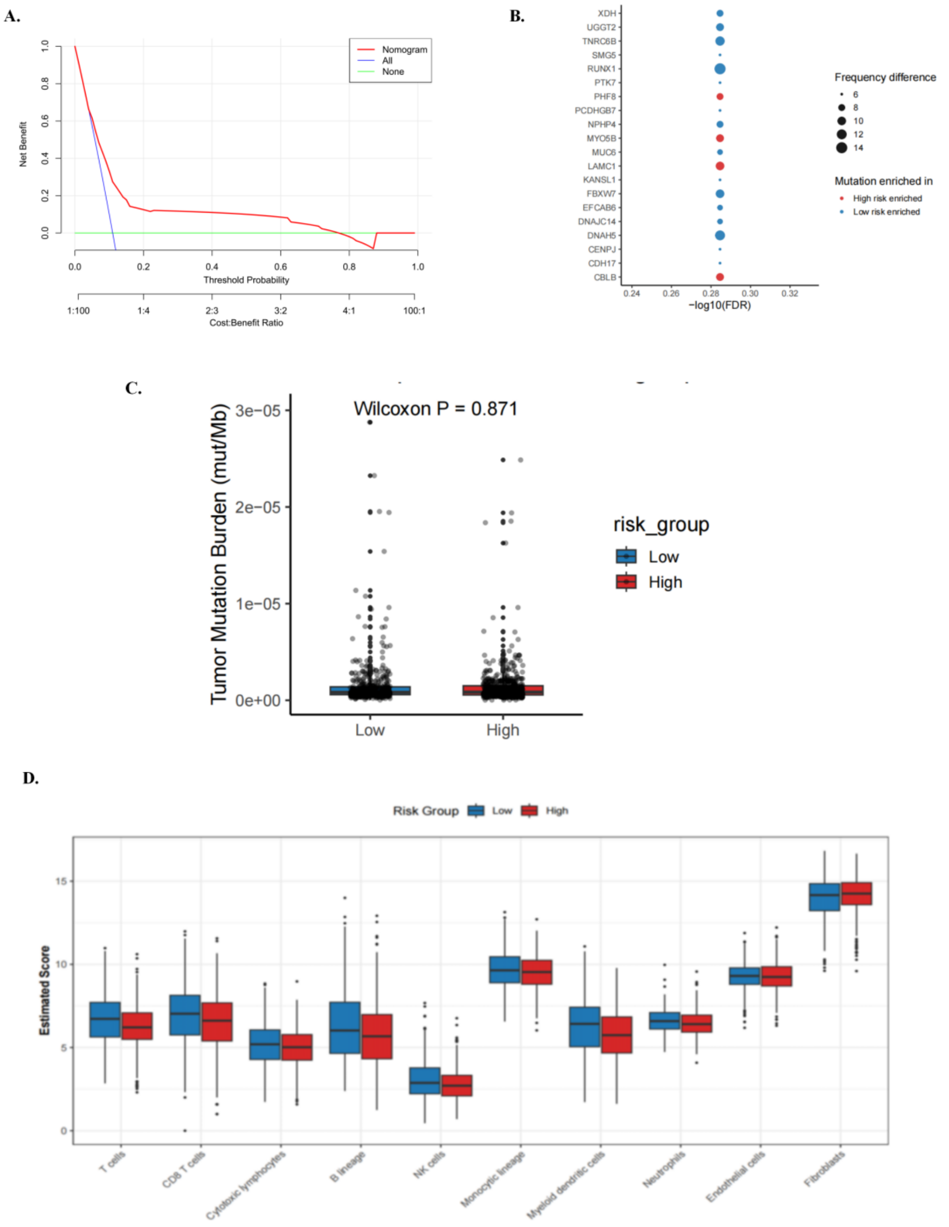

Supplementary Figure 5. Additional clinical utility, genomic, and immune-stromal validation analyses of the prognostic model. (A) Decision curve analysis (DCA) of the nomogram model. (B) Differential mutation-frequency analysis between the high-risk and low-risk groups. Dot size indicates the magnitude of mutation-frequency difference, and colour indicates the group in which

the mutation is enriched. (C) Comparison of tumour mutation burden (TMB) between the high-risk and low-risk groups. (D) MCP-counter analysis showing differences in estimated immune/stromal features between the low-risk and high-risk groups.
