# Supplementary material for: Lactylation-mediated remodelling of the breast cancer microenvironment: single-cell multidimensional analysis and prognostic model construction
Source: Front Immunol. 2026 May 13;17:1747043. doi: 10.3389/fimmu.2026.1747043 (PMC13212231; doi:10.3389/fimmu.2026.1747043)
Supplement: Supplementary Figure 1 — Single-cell transcriptome subtype analysis and PPI network construction of breast cancer. (A) The UMAP dimensionality reduction map shows the clustering of cell clusters. (B) Stratified cell type statistics by molecular subtypes (ER+, HER2+ and TNBC). (C) UMAP view of lactic acid activity stratified by molecular subtypes (ER+, HER2+, and TNBC), highlighting the lactic acid expression of subtypes. (D) The expression of lactic acid activity stratified by molecular subtypes (ER+, HER2+ and TNBC) in each cell type. (E) PPI network diagram of prognostic genes. (F) Box plot of expression differences of prognostic genes in tumours and normal tissues. [file DataSheet1.zip › 实验原始数据/IHC Statement.DOCX]

**Original Image Statement**

All immunohistochemistry (IHC) images presented in this manuscript are original, unprocessed images. No cropping beyond panel layout, no alteration of colour balance, and no digital enhancement that would affect data interpretation have been applied. The images used in the figures were taken directly from the original microscopy files, and full-resolution raw image data are available from the corresponding author upon reasonable request.
