# Supplementary figures and images for: Lactylation-mediated remodelling of the breast cancer microenvironment: single-cell multidimensional analysis and prognostic model construction
Source: Front Immunol. 2026 May 13;17:1747043. doi: 10.3389/fimmu.2026.1747043 (PMC13212231; doi:10.3389/fimmu.2026.1747043)

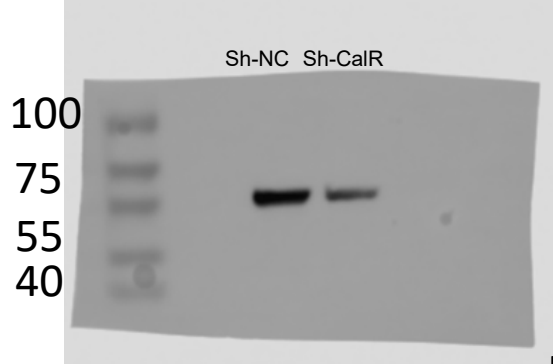

IB:CalR

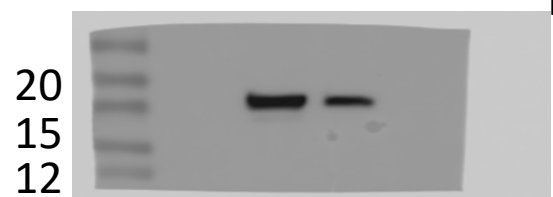

IB:Cyclin D1

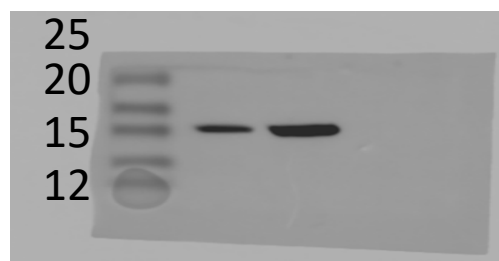

IB:CDK2B

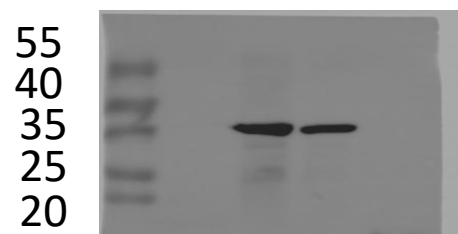

IB:CDK4

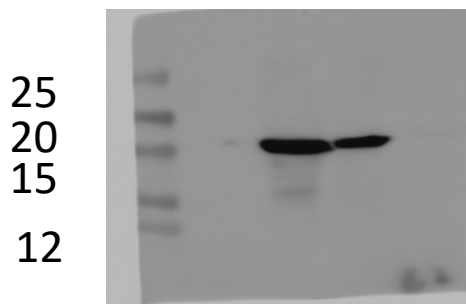

IB:CDK2C

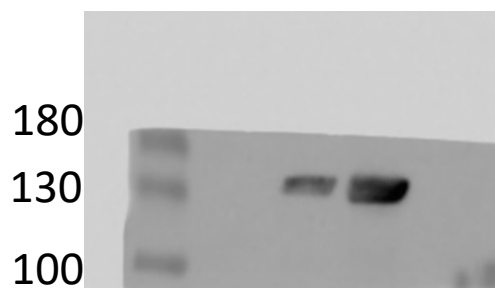

IB:pRB1

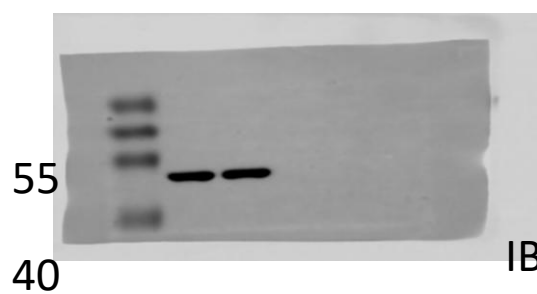

IB: $\beta$ -Actin

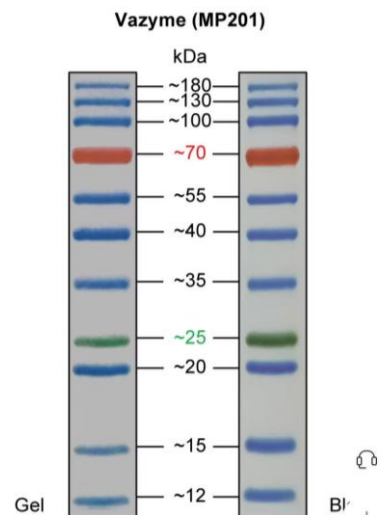

shNC shCALR  
#1 #2

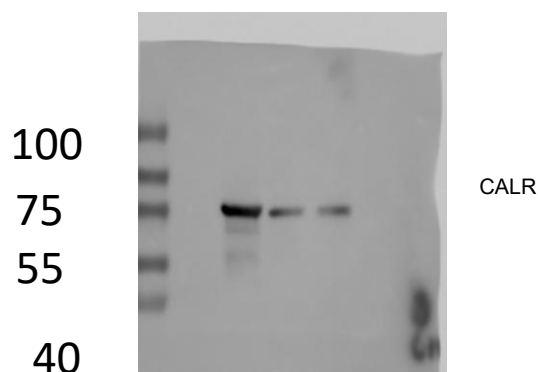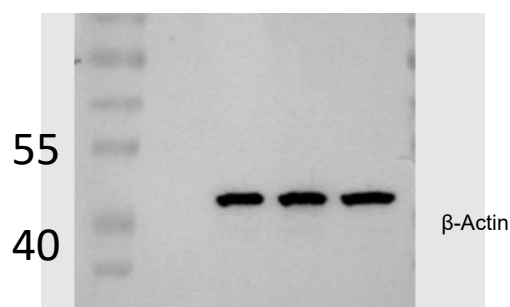

Supplement: Supplementary Figure 1 — Single-cell transcriptome subtype analysis and PPI network construction of breast cancer. (A) The UMAP dimensionality reduction map shows the clustering of cell clusters. (B) Stratified cell type statistics by molecular subtypes (ER+, HER2+ and TNBC). (C) UMAP view of lactic acid activity stratified by molecular subtypes (ER+, HER2+, and TNBC), highlighting the lactic acid expression of subtypes. (D) The expression of lactic acid activity stratified by molecular subtypes (ER+, HER2+ and TNBC) in each cell type. (E) PPI network diagram of prognostic genes. (F) Box plot of expression differences of prognostic genes in tumours and normal tissues. [file DataSheet1.zip › 实验原始数据/WB with marker original picture.pdf]
